# Treatment options for digital nerve injury: a systematic review and meta-analysis

**DOI:** 10.1186/s13018-023-04076-x

**Published:** 2023-09-12

**Authors:** Yi Zhang, Nianzong Hou, Jian Zhang, Bing Xie, Jiahui Liang, Xiaohu Chang, Kai Wang, Xin Tang

**Affiliations:** 1 Department of Orthopedics, First Affiliated Hospital of Dalian Medical University, Dalian, 116011 Liaoning China; 2 Department of Hand and Foot Surgery, Zibo Central Hospital, No. 54 Gongqingtuan West Road, Zibo, Shandong China; 3 Department of Critical Care Medicine, Zibo Central Hospital, No. 54 Gongqingtuan West Road, Zibo, Shandong China; 4 Center of Gallbladder Disease, Shanghai East Hospital, Institute of Gallstone Disease, School of Medicine, Tongji University, Shanghai, China

**Keywords:** Digital nerve, Digital nerve injury, Digital nerve repair, Digital nerve reconstruction, Digital nerve gap repair

## Abstract

**Background:**

Surgical treatment of finger nerve injury is common for hand trauma. However, there are various surgical options with different functional outcomes. The aims of this study are to compare the outcomes of various finger nerve surgeries and to identify factors associated with the postsurgical outcomes via a systematic review and meta-analysis.

**Methods:**

The literature related to digital nerve repairs were retrieved comprehensively by searching the online databases of PubMed from January 1, 1965, to August 31, 2021. Data extraction, assessment of bias risk and the quality evaluation were then performed. Meta-analysis was performed using the postoperative static 2-point discrimination (S2PD) value, moving 2-point discrimination (M2PD) value, and Semmes–Weinstein monofilament testing (SWMF) good rate, modified Highet classification of nerve recovery good rate. Statistical analysis was performed using the R (V.3.6.3) software. The random effects model was used for the analysis. A systematic review was also performed on the other influencing factors especially the type of injury and postoperative complications of digital nerve repair.

**Results:**

Sixty-six studies with 2446 cases were included in this study. The polyglycolic acid conduit group has the best S2PD value (6.71 mm), while the neurorrhaphy group has the best M2PD value (4.91 mm). End-to-side coaptation has the highest modified Highet’s scoring (98%), and autologous nerve graft has the highest SWMF (91%). Age, the size of the gap, and the type of injury were factors that may affect recovery. The type of injury has an impact on the postoperative outcome of neurorrhaphy. Complications reported in the studies were mainly neuroma, cold sensitivity, paresthesia, postoperative infection, and pain.

**Conclusion:**

Our study demonstrated that the results of surgical treatment of digital nerve injury are generally satisfactory; however, no nerve repair method has absolute advantages. When choosing a surgical approach to repair finger nerve injury, we must comprehensively consider various factors, especially the gap size of the nerve defect, and postoperative complications.

*Type of study/level of evidence* Therapeutic IV.

## Background

Finger nerve laceration is one of the most common injuries in hand trauma, and its incidence rate is high in the peripheral nerve injuries of the upper limbs [1]. Most hand injuries with nerve damage require surgical treatment [2]. Potential common complications from either surgical or non-surgical treatments include numbness, paresthesia, neuroma, and cold intolerance [3].

Finger nerve repair currently has two main surgical approaches. End-to-end tension-free neurorrhaphy has traditionally been the preferred repair method in lesions with a gap smaller than 5 mm [2]. When the nerve ends cannot be approximated without tension, nerve reconstruction becomes the most commonly used method. [4] Various materials are available for reconstruction, such as autograft, nerve autograft, nerve allograft, and artificial conduit. End-to-side anastomosis is also commonly used to reconstruct large nerve defects. The repair materials of autograft mainly include veins and muscle-in-vein [5]. The autologous nerve graft is the historical gold standard for nerve reconstruction [2]. However, the autologous nerve graft damages the patient’s own tissue, which can increase operative time for harvesting donor nerve and increase potential donor site morbidity [6]. With the improvement of technology and repair materials, nerve duct repair technology and allogeneic nerve repair technology are now available. These two techniques avoid donor site complications caused by autologous nerve transplantation [5]. Synthetic nerve conduits have polyglycolic acid (PGA) tubes and collagen tubes. However, potential complications of allogeneic transplantation include the transmission of infectious diseases [5]. For large-segment defects or proximal nerve damage, some scholars have tried the technique of end-to-side nerve anastomosis. This method can bridge the damaged nerve to the healthy nerve [7].

In addition to the surgical method that may affect the functional outcomes, other predictors of sensory recovery have been evaluated in several studies, such as mechanism of injury gender, age, involved digit, level of injury, time from injury till repair, and gap length. The main one is the type of injury, which can affect the severity of the nerve damage, the gap between the nerve defects, and the recovery after surgery. According to Kusuhara et al. [8], avulsion injuries had significantly lower levels of meaningful recovery when compared with those of clean-cut and crush types of injury. However, Schmauss et al.’s study [9] suggested that it did not observe significant differences in sharp versus crush injuries.

Few systematic reviews and meta-analyses have been conducted to compare surgical approaches and factors associated with sensory outcomes of digital nerve repair. [2, 3, 5, 10–13] In 2013 Paprottka et al.’s research, some of the included studies were low quality, and they did not compare allogeneic nerve repairs [5]. Herman et al. and Mauch et al.’s research in 2019 [8] included fewer studies and performed limited subgroups analyzed due to small sample size [2, 10]. Thus, we aimed to perform a comprehensive meta-analysis and systematic review of finger nerve repair to include high-quality studies with large sample sizes and conduct detailed subgroup analysis to compare different surgical approaches. We also aimed to identify factors associated with the functional outcomes of finger nerve repair.

## Methods

We performed and reported this review based on the Preferred Reporting Items for Systematic Reviews and Meta-Analyses (PRISMA) guidelines.

### Search strategy and inclusion/exclusion criteria

We performed systematic literature search in PubMed. The search terms “digital nerve,” “operation,” “surgery,” “nerve injury,” “nerve repair,” were combined using Boolean operators. Both “free-text term” and “MeSH term” searches were completed. We did not impose any restrictions on the language. The publication date was set from January 1, 1965, to August 31, 2021, because the clinical implementation of the surgical microscope started around 1965. The previous surgeries without microscopes were not included in the study [14]. Additionally, we reviewed the reference lists of the included papers and previously published reviews to ensure relevant studies had been considered. We merged all search results and discarded duplicate citations [2, 3, 5, 10–13].

**Fig. 1:**
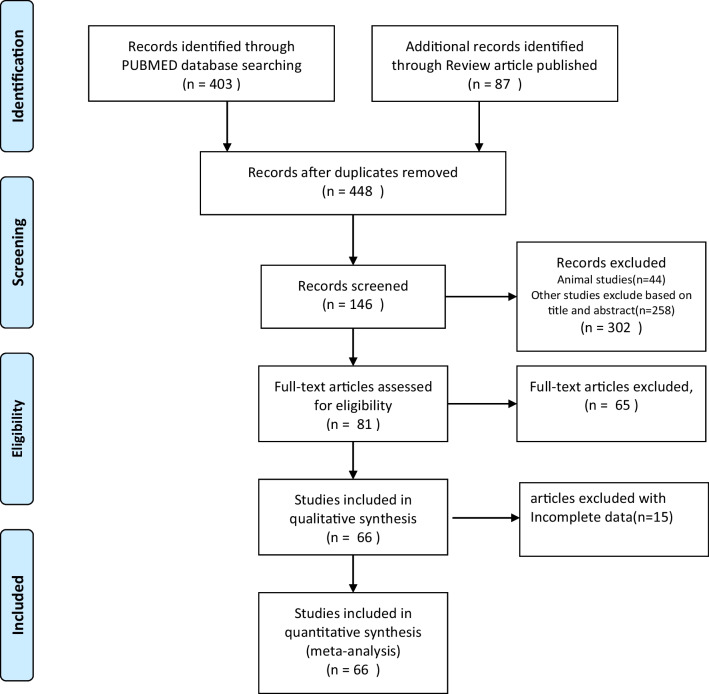
Flowchart of studies identified, included, and excluded

Two authors screened the articles independently based on the titles and abstracts, and each author independently retrieved and examined the full texts of the relevant papers for inclusion/exclusion based on predefined stratified criteria. Finally, we included all prospective and retrospective studies on surgical treatment of finger nerve injuries, including observational cohort studies, randomized controlled trials, and case reports with detailed data. We included patients of all ages with finger nerve injuries. The data published on the included studies were analyzed for the outcomes. We included results with at least 6-month follow-up. Exclusion criteria were peripheral nerve lesions not localized to the digital nerves in the hand, duplicated data, without appropriate data analysis methods, inconsistent data, reviews, unpublished literature, conference papers, studies without adequate information. The PRISMA flowchart is shown in Fig. 1.

### Data extraction and outcome measures

The primary author extracted data onto a predefined electronic data extraction form, and then, the other author checked all the data. Any disagreements were resolved through discussion, if necessary, with the involvement of a third reviewer. We extract the following data from each included literature, the characteristics of the literature (author, nationality, research type, hospital, date), population characteristics (age, gender, sample size, number of lost follow-up, number of injured nerves, smoking, type of injury), damage and repair status (nerve gap, repair time, type of surgery, follow-up time), complications (postoperative neuroma, cold stimulation, paresthesia, postoperative infection, pain).

The outcome measurements we used included: static 2-point discrimination (S2PD), moving 2-point dis crimination (M2PD), Semmes–Weinstein monofilament testing (SWMF), and modified Highet classification of nerve recovery [3]. Weber first described S2PD in 1835 which was the most widely used outcome measure. Normal values of S2PD in an uninjured fingertip range from 2 to 6 mm. M2PD was described by Dellon, and we used it as the second outcome indicator to evaluate the recovery of the finger nerves after surgery. S2PD and M2PD use actual measurement distance to evaluate the degree of nerve recovery. They are both continuous variables. The shorter the measurement distance, the better the response.

We used a modified classification system derived from Imai et al. to group SWMF outcomes. The SWMF scores ≤ 2.83 mean “normal” for sensation, scores from 2.83 to 4.31 mean “diminished light touch,” scores from 4.31 to 4.56 mean “diminished protective sensation,” scores from 4.56 to 6.10 mean “loss of protective sensation,” and scores > 6.10 mean “anesthetic” [15]. We counted the number of people with a score less than 4.31 (full sensation and diminished light touch) to calculate the excellent rate for the degree of recovery.

Medical Research Council scoring system from 1954, modified by MacKinnon and Dellon often referred to as modified Highet, grouped a range of values into subjective headings [3]. This scoring system was often used to evaluate the recovery after nerve repair. The specific evaluation criteria are shown in Table 1. We extracted the sensory recovery as good and excellent nerve numbers in the table to evaluate the effect of the treatment.

**Table 1:** Modified Highet classification of nerve recovery

Sensory recovery	Highet	s2PD	m2PD	Recovery of sensibility Sensory recovery
Failure	S0			No recovery of sensibility in the autonomous zone of the nerve
Poor	S1	> 15 mm	> 7 mm	Recovery of deep cutaneous pain sensibility
	S1 +			Recovery of superficial pain sensibility
	S2			Recovery of superficial pain and some touch sensibility
	S2 +			As with S2, but with over response
	S3			Recovery of pain and touch sensibly with no over response
Good	S3 +	7–15 mm	4–7 mm	As in S3, but with good localization of the stimulus but imperfect recovery of 2PD
Excellent	S4	2–6 mm	2–3 mm	Complete sensory recovery

In the S2PD and Highet data sets, there were many accounting articles, large amounts of data, and more detailed data. Therefore, we divided artificial catheters into two subgroups: collagen tubes and polyglycolic acid catheters. We divided venous catheters and muscle-in-vein grafts into groups in the autograft method. Direct suture and end-to-side anastomosis were split into two subgroups of neurorrhaphy for analysis. For these two data groups, we divided them into artificial conduit: polyglycolic acid, artificial conduit: collagen, nerve allograft, autograft repair: muscle-in-vein graft, autograft repair: vein graft, autologous nerve graft, end-to-end coaptation, end-to-side coaptation, total 8 repair types.

There were fewer articles in the M2PD and SWMF data sets, so the data we extracted were limited. When summarizing and analyzing the data, we did not conduct a detailed subgroup analysis but merged them into five repair Types for analysis. They were: artificial conduit (collagen tubes/polyglycolic acid catheters), nerve allograft, autograft repair (muscle-in-vein graft/vein graft), autologous nerve graft, and neurorrhaphy (end-to-end coaptation/end-to-side coaptation).

In addition, to evaluate the outcomes of the surgical repair methods, we also summarized and analyzed other factors associated with the result. These factors mainly included age, never gap, injury type, repair time, and smoking. Of course, the most important of these factors is the type of injury, which affects the degree of nerve damage, the choice of the surgical method, and postoperative recovery. We analyzed 25 articles [1, 7, 16–38] with specific injury descriptions through further screening of the included literature. We divided the injury types into sharp injury and crush injury. Sharp injuries include cutting injuries, acute or semi-sharp injuries, and stab injuries. Crush injuries include serious crush injuries, mangled injuries, and lacerated injuries. We analyzed patients with two types of injury in four types of surgery, and the analysis indexes were S2PD and modified Highet score excellent rate.

Complications reported in the studies were mainly neuroma, cold sensitivity, paresthesia, postoperative infection, and pain. We also conducted a summary analysis.

### Statistical analysis, risk of bias, and study quality assessment

Our meta-analysis was performed by R (V.3.6.3) and package of meta. Heterogeneity variance parameter I^2^ test was used to assess the heterogeneity of the model. However, in order to reduce the difference between the parameters and avoid error of the results caused by heterogeneity, the random effects model was used to merge the statistics. For postoperative S2PD and M2PD of various surgical methods, we use a combined statistical analysis of mean and standard deviation. For the SWMF excellent rate and modified Highet score excellent rate, we adopted a combined statistical analysis of the rates. The results of the merger were displayed in a forest diagram, and the statistics were compared in the form of a table. We used funnel chart and egger test for publication bias. In the analysis by surgical method and injury type, the continuous variables of S2PD were compared by T test, and the excellent and good rates were compared using the chi-square test.

We used standardized critical appraisal instruments from the JBI Meta-Analysis of Statistics Assessment and Review Instrument (JBI-MAStARI) (Appendix II) to evaluate all included literature. Because all the included studies were case series or cohort studies, we used JBI Critical Appraisal Checklist for Descriptive/Case Series to evaluate the quality of the literature. This evaluation checklist includes 9 quality items, and the judging options include yes, no, unclear, and not applicable. Studies that blinded the evaluators and had “yes” scores of 80% were considered high quality; those with “yes” scores of 60–80% were rated as medium, and the quality of studies with a score of less than 60% was considered low. Any disagreements that arose between the reviewers were resolved through discussion.

## Results

### Study selection

We searched the PubMed database using keywords and got 403 different publications. At the same time, we examined the reference lists of the included papers and previous reviews to add 45 records. Sixty-six articles were included in the final data analysis [1, 7–9, 16–76, 86] (Fig. 1).

### Study characteristics

The 66 articles included a total of 2446 cases. Fifty studies [1, 7, 16, 19, 21, 25–39, 41, 42, 45–52, 59–76, 86] were retrospective case series, and 16 [8, 9, 17, 18, 20, 22–24, 40, 43, 44, 53–57] were prospective. Of these studies, 16 control studies were available [20, 21, 28, 29, 38, 40–42, 53–60]. There were 3 papers that we only extracted part of the data because they included other nerve injuries in addition to the finger nerves [7, 32, 61]. The age range of patients included in these studies was 1–81 years old. The time from injury to surgical repair ranged between 0 and 37 months, and follow-up time ranged between 6 and 202 months. The detailed characteristics of eligible studies are shown in Table 2.

**Table 2:** The characteristics of included studies

Article	Country	Study type	Research institute	Date	Intervention (s) and control	Number of digital nerve repairs analyzed	Number of people lost to follow-up	Gender (M/F)	Never gap mean (range)
Kusuhara et al. [8]	Japan	Prospective	Faculty of Medicine, Kindai University	2013.07 to 2016.05	PGA	20	0	M15/F5	16.7 mm (1–50 mm)
Mackinnon and Dellon [39]	USA	Retrospective	University of Toronto Sunnybrook Medical Center	1985.12–1988.10	PGA	15	0	–	1.7 cm (0.5–3.0 cm)
Rinker and Liau [40]	USA	Prospective	University of Kentucky, Lexington, KY	–	PGA VS Vein graft;(A/B)	56 (A:34; B:22)	5 (12%)	M29/F8	10 mm (4–25 mm)
Battiston et al. [41]	Italy	Retrospective	C.T.O. Hospital, Turin, Italy	1998.06–2002.12	PGA VS Vein graft; A/B	32 (A:19; B:13)	0	–	A:2 cm (1–4 cm); B:1.1 cm (0.5–1.5 cm)
Neubrech et al. [42]	Germany	Retrospective	BG Unfallklinik Ludwigshafen	2009–2013	PGA VS end-to-end coaptation; A/B	38 (A:15; B:23)	–	M27/F14	2–3 cm
Bushnell et al. [16]	USA	Retrospective	University of North Carolina Hospitals	2005.01–2006.12	Collagen conduit	9	3 (25%)	M8/F4	10–20 mm
Lohmeyer et al. [43]	Germany	Prospective	Clinic on the right Isar, Technical University of Munich	Over a period of 3 years	Collagen conduit	40	9 (18%)	M45/F4	12 mm (5–25 mm)
Lohmeyer et al. [44]	Germany	Prospective	University Hospital Schleswig–Holstein	2004.07–2006.05	Collagen conduit	6	5 (45%)	M7/F4	18 mm
Schmauss et al. [9]	Germany	Prospective	Klinikum rechts der Isar, Technische Universität München	2004.07–2006.11 and 2007.05–2011.09	Collagen conduit	20	29 (64%)	M12/F4	11.0 mm (6–25 mm)
Taras et al. [17]		Prospective	Thomas Jefferson University	2002.09–2007.01	Collagen conduit	22	–	M10/F12	12 mm (5–17 mm)
Arnaout et al. [18]	France	Prospective	centre hospitalier régional universitaire	2009.11–2010.04	Collagen conduit	27	11 (31%)	M20/F4	–
Thomsen et al. [19]	France	Retrospective	San Antonio University Hospital	2007.11–2008.10	Collagen conduit	11	4 (22%)	M3/F7	11.25 mm (5–20 mm)
Means et al. [20]	USA	Prospective	Curtis National Hand Center	–	Collagen conduit VS nerve allograft; A/B	15 (A6: B9)	11 (48%)	M18/F5	12 mm (5–20 mm)
Rbia et al. [21]	USA	Retrospective	Mayo Clinic, Minnesota, USA	from 2007 to 2015	Collagen conduit VS nerve allograft; A/B	37 (A:19, B:18)	–	M34/F3	A:14 ± 4.9 mm; B: 18.4 ± 9.3 mm
Guo et al. [22]	China	Prospective	Beijing Jishuitan Hospital	2009.10–2010.07	Nerve allograft	5	0	M5	23 mm (18–28 mm)
Ingari [45]	USA	Retrospective	University of Kentucky College of Medicine	–	Nerve allograft	37	0	M21/F3	11 mm (5–15 mm)
Rinker [46]	USA	Retrospective	University of Kentucky, etc.	–	Nerve allograft	50	0	M22/F6	35 mm (25–50 mm)
Taras [23]	USA	Prospective	The Thomas Jefferson University, etc.	–	Nerve allograft	18	3 (18%)	M10/F4	11 mm (5–30 mm)
Karabekmez et al. [24]	USA	Prospective	Division of Plastic Surgery, Mayo Clinic, Rochester, MN, USA	2007.07–2008.03	Nerve allograft	10	0	M5/F2	2.23 cm (0.5–3 cm)
He [53]	China	Prospective	The First Affiliated Hospital of Sun Yat-sen University	–	Nerve allograft VS end-to-end coaptation; A/B	218 (A: 95 B: 123)	5 (3%)	A:M67/F28; B:M73/F50	1.80 cm (1–5 cm)
Ignazio [47]	Italy	Retrospective	Istituto Clinico Città di Brescia–Gruppo San Donato	–	Muscle-in-vein graft	21	0	M11/F6	2.2 cm (1–3.5 cm)
Norris et al. [48]	Britain	Retrospective	Queen Victoria Hospital, East Grinstead, and the Royal College of Surgeons of England	–	Muscle-in-vein graft	8	0	M6/F2	15–25 mm
Tos et al. [25]	Italy	Retrospective	CTO-M. Adelaide Hospital	1995 to 2008	Muscle-in-vein graft	8	0	M12/F4	1.2 cm (0.5–1.5 cm)
Pereira et al. [38]	Britain	Retrospective	Royal College of Surgeons of England and The Queen Victoria Hospital	–	Muscle-in-vein graft VS end-to-end coaptation; A/B	A: 12; B: 29	0	A:M6/F4; B:M20/F4	–
Laveaux et al. [49]	France	Retrospective	University of Franche-Comté, Besançon	–	Vein graft	12	0	M11/F1	15 mm (3–25 mm)
Lee and Shieh [50]	China, Taiwan	Retrospective	National Cheng Kung University Medical Center	From 1995 to 2005	Vein graft	3	0	M3	1.43 cm (0.8–2.5 cm)
Risitano et al. [26]	Italy	Retrospective	University of Messina, Italy	–	Vein graft	22	0	–	1.39 cm (1–3 cm.)
Tang et al. [51]	China	Retrospective	Affiliated Hospital of Nantong Medical College	1990.01–1991.05	Vein graft	18	0	M9/F6	2 cm (0.5–5.8 cm)
Walton et al. [52]	USA	Retrospective	University of Massachusetts Medical Center	1985.08–1987.04	Vein graft	18	1 (7%)	M9/F5	1.7 cm (1–3 cm)
Chiu and Strauch [54]	USA	Prospective	he experimentation committees of New York University Medical Center and the Cabrini Medical Center	1982–1988	End-to-end coaptation VS Vein graft VS autologous nerve graft; A/B/C	26 (A:12; B:10; C:4)	2	–	< 3 cm
Calcagnotto and Braga Silva [57]	Brazil	Prospective	Service de chirurgie de la main, hôpital da PUC	–	Autologous nerve graft VS Vein graft; A/B	50 (A:25 B:25)	–	A:M22/F3; B:M25/F0	A:15.3 ± 3.8 mm B:14.6 ± 5.5 mm
Alligand-perrin et al. [27]	France	Retrospective	–	–	Vein graft	53	0	M28/F20	–
Laveaux et al. [28]	France	Retrospective	Franche Comte University		Vein graft VS autologous nerve graft; A/B	32 (A:17; B:15)	0	A:M14/F3 B:M11/F4	A:17 mm (5–30 mm) B:18 mm (10–30 mm)
Rose et al. [68]	USA	Retrospective (case report)	the Stanford University School of Medicine, etc.	–	Autologous nerve graft	14	0	M9/F1	4.4 cm (2.5–9.0 cm)
Chen et al. [29]	China	Retrospective	the Affiliated Hospital of North China Coal Medical College	2005.05 to 2010.03	Autologous nerve graft with artery VS autologous nerve graft; A/B	A:16; B:27	0	M13/F3	2.5 cm
Li et al. [86]	China	Retrospective	Renqiu City People’s Hospital	2007.01–2015.05	Autologous nerve graft	23	0	M17/F6	–
Chen et al. [58]	China	Retrospective	the Affiliated Hospital of North China Coal Medical College	2005.02–2009.07	End-to-side coaptation VS Autologous nerve graft; A/B	A:21; B:31	0	A:M14/F3; B:M25/F6	A:2.3 cm (1.4–3.5 cm); B:2.4 cm (1.4–3.6 cm)
Stang et al. [62]	Germany	Retrospective	University Hospital Schleswig–Holstein	2006–2007	Autologous nerve graft (posterior interosseous nerve VS the medial antebrachial cutaneous nerve; A/B)	28	0	A:M13/F3; B:M10/F2	A:22 ± 5 mm; B:22 ± 11 mm
Chevrollier et al. [67]	France	Retrospective	Emile Galle surgical center, Nancy	2008–2012	Autologous nerve graft	16	8 (33%)	M13/F3	38 mm (15–60 mm)
Kim et al. [69]	South Korea	Retrospective	Seoul National University College of Medicine	2006.03–2012.02	Autologous nerve graft	30	0	M22/F3	–
Mcfarlane and Mayer [65]	Canada	Retrospective	the University of Western Ontario and Victoria Hospital	–	Autologous nerve graft	13	0	–	2 cm (1.5–3.5 cm)
Nunley et al. [76]	USA	Retrospective	Duke University Medical Cent	1977.12–1982.08	Autologous nerve graft	21	0	M8/F6	2.5 cm (1.5–4 cm)
Pilanci et al. [30]	Turkey	Retrospective	Bagcilar Research and Training Hospital	2009.02–2012.09	Autologous nerve graft	15	0	M13/F2	1.81 cm (0.75–3 cm)
Bekir [71]	Turkey	Retrospective	Göztepe Medicalpark Hospital	2007–2012	Autologous nerve graft	13	0	M9/F2	18.5 mm (15–25 mm)
Inoue et al. [31]	Japan	Retrospective	Yamagata University	1993–1998	Autologous nerve graft	3	0	M2/F1	1.3 cm (1–1.5 cm)
Young et al. [55]	USA	Retrospective	Washington University School of Medicine	1974–1978	Autologous nerve graft	33	6	M30/F8	–
Meek et al. [32]	Netherlands	Retrospective	University Hospital Groningen	–	Autologous nerve graft	17	–	M31/F10	–
Acar et al. [75]	Turkey	Retrospective	Konya Necmettin Erbakan University Meram School of Medicine	2012.01 and 2014.11	End-to-end coaptation	138	0	M42/F6	–
Alghazal [33]	Ireland	Retrospective	University College Hospital, Galway	1989.09 and 1991.09	End-to-end coaptation	88	0	M58/F26	–
Altissimi et al. [34]	Italy	Retrospective	University of Perugia	In a six-year period,	End-to-end coaptation	54	–	M32/F8	–
Efstathopoulos et al. [1]	Greece	Retrospective	“K.A.T.” Accident Hospital	1988 and 1993	End-to-end coaptation	64	–	M42/F8	–
Fakin et al. [63]	Switzerland	Retrospective	University Hospital Zurich	2006.06–2011.12	End-to-end coaptation	93	–	M56/F27	–
Poppen et al. [66]	USA	Retrospective	the Pacific Medical Center	1963.01 and 1972.07	End-to-end coaptation	74	49 (50%)	M31/F18	–
Sladana et al. [72]	Serbia	Retrospective	Clinical Center of Serbia	2005.01 and 2009.12	End-to-end coaptation	193	–	M126/F24	–
Sullivan [35]	USA	Retrospective	the Tripler Army Medical Center, Hawaii	–	End-to-end coaptation	43	–	–	–
Bulut et al. [73]	Turkey	Retrospective	Atatürk Training and Research Hospital	2009.01 and 2013.07	End-to-end coaptation	96	–	–	–
Oruç et al. [59]	Turkey	Retrospective	Ankara Training and Research Hospital	2013.01 and 2014.12	End-to-end coaptation (Unilateral VS Bilateral; A/B)	28 (A 18; B 10)	–	A:10 M/F2; B:5 M/F2	–
Young et al. [51]	USA	Prospective	Washington University School of Medicine	1977.01–1979.08	End-to-end coaptation (fascicular VS epineural; A/B)	34 (A:17; B:17)	–	M18/F9	–
Segalman et al. [36]	USA	Retrospective	Union Memorial Hospital	Over an 8-year period	End-to-end coaptation (old people)	19	–	M7/F8	–
Vahvanen et al. [74]	Finland	Retrospective	Aurora Hospital, Helsinki, Finland	1961–1977	End-to-end coaptation	18	5 (12%)	M32/F11	–
Wang et al. [60]	USA	Retrospective	The Christine M. Kleinert Institute for Hand and Micro Surgery	1984.01 to 1991.12	End-to-end coaptation VS autologous nerve graft; A/B	90 (A:76; B:14)	0	–	1.5–6 cm
Mennen [84]	South Africa	Retrospective	Medical University of Southern Africa	1996 to 2000	End-to-side coaptation	5	–	M5/F0	–
Voche and Ouattara [37]	France	Retrospective	Clinique La Francilienne	1999.10 to 2003.06	End-to-side coaptation	11	2 (17%)	M7/F3	2.5 cm (1.5 –4 cm)
Landwehrs and Brüser [70]	Germany	Retrospective	Malteser Krankenhaus Bonn/Rhein-Sieg, Bonn	2002.04–2004.07	End-to-side coaptation	5	0	–	–
Artiaco et al. [64]	Italy	Retrospective	University of Turin Studies, Italy	2002–2008	End-to-side coaptation	7	1 (13%)	M4/F3	–
Chow and Ng [56]	Hong Kong, China	Prospective	University of Hong Kong	1986.11 to 1988.12	End-to-end coaptation (neurorrhaphy VS non-neurorrhaphy; A/B)	A:72; B:36	47 (36%)	M66/F19	–

Article	Age (years) mean (range)	Follow-up time mean (range)	Static 2-point discrimination	Moving 2-point discrimination	SWMF testing (full sensation)	Modified Highet (S3 + ,S4) (good)	Repair time (time after injury)	Quality score (JBI Critical Appraisal Checklist, cf. Table 3)
Kusuhara et al. [8]	47 Y (18–79 Y)	13 mth	8.6 ± 1.2 mm	–	–	18 (90%)	–	High
Mackinnon and Dellon [39]	30.5 ± 7.6 Y	22.4 mth (11–32 mth )	4.6 ± 1.1 mm	3.3 ± 0.7 mm	–	8 (53%)	–	High
Rinker and Liau [40]	35Y (19–76 Y)	A:12 mth ; B:12 mth	A:7.5 ± 1.9 mm B:7.6 ± 2.6 mm	A:5.6 ± 2.2 mm B:6.6 ± 2.9 mm	–	–	A: 18 ± 6 Minutes B: 34 ± 9 min	High
Battiston et al. [41]	A:40 Y (15–67 Y) B:35.6 Y (20–50 Y)	6–74 mth	–	A: 9.6 ± 4.1 mm B: 8.2 ± 4.2 mm	–	A:13 (68%) B:10 (77%)	1–16 mth	High
Neubrech et al. [42]	42 Y (14–71 Y)	34 mth (10-76 mth)	A: 5.5 mm (3–15; SD: 5) B: 4.5 mm (3–15; SD: 3.9)	–	–	–	–	High
Bushnell et al. [16]	33 Y (18–50 Y)	15 mth (12–22 mth)	6.9 ± 2.9 mm	–	5 (56%)	4 (44%)	–	High
Lohmeyer et al. [43]	37.9 Y (17–75 Y)	12 mth	–	–	–	20 (50%)	95 days (19–264 days)	High
Lohmeyer et al. [44]	36.7 Y (12–66 Y)	12 mth	8.3 ± 5.3 mm	–	–	5 (83%)	0–37 mth	High
Schmauss et al. [9]	40.0 Y (20–75 Y)	58.1 mth (29.3–93.3 mth)	6.8 mm (3–15 mm)	–	8 (40%)	17 (85%)	–	High
Taras et al. [17]	44 Y (22–72 Y)	20 mth (12–59 mth)	5.2 ± 1.5 mm	5 ± 1.7 mm	–	–	6 day (1–19 day)	High
Arnaout et al. [18]	38 Y (13.5–71 Y)	6 mth	10.3 ± 3.76 mm;		12 (45%)	23 (85%)	< 24 hs	High
Thomsen et al. [19]	30 Y (16–49 Y)	11.8 mth (6–17 mth)	9.6 ± 4.9 mm		9 (82%)	10 (91%)	5.9 mth (3–11 mth)	High
Means et al. [20]	41 Y (20–63 Y)	12 mth	A: 5 ± 1 mm; B: 8 ± 5 mm	A: 5 ± 1 mm; B: 7 ± 5 mm	A:6 (100%); B:7 (78%)	A:6 (100%); B:7 (78%)	< 12 weeks	High
Rbia et al. [21]	A; 38 Y (9–72 Y) B:41 (12–62 Y)	< 12 mth	A: 9.8 ± 3.8 mm B: 8.5 ± 3.7 mm	–	–	A:14 (74%); B:17 (94%)	< 3 weeks	High
Guo et al. [22]	33 Y (18–39 Y)	12 mth	6 ± 0.6 mm	–	3 (60%)	5 (100%)	Within 8 hs	High
Ingari [45]	43 Y (23–81 Y)	13 days (0–215) days	7.1 ± 2.9 mm (*n* = 19)	6.7 ± 3.3 mm (*n* = 17)	23 (72%) (*n* = 32)	31 (84%)	13 (0–215) days	High
Rinker [46]	45 Y (22–78 Y)	11 mth	9 ± 4 mm	–	33 (87%) (*n* = 38)	32 (64%)	6 (0–2514) days	High
Taras [23]	39 Y (18–76 Y)	15 mth (> 12 mth)	7.1 ± 1.1 mm	5.4 ± 1.8 m	14 (78%)	14 (78%)	–	High
Karabekmez et al. [24]	44 Y (23–65 Y)	9 mth (5–12 mth)	5.5 ± 2.5 mm (*n* = 8)	4.4 ± 1.1 mm (*n* = 10)	–	10 (100%)	–	Medium
He [53]	A:33 Y (18–61 Y); B:36.9 Y (15–77 Y)	6 mth	A: 12.81 ± 5.99 mm	–	A: 90 (95%); B: 113 (92%)	A:68 (72%); B:73 (59%)	A:23.7 days (0–200 days); B:1.5 day (0–91 days)	High
Ignazio [47]	38 Y (11–61 Y)	> 18 mth (mean, 43 mth; 18–96 mth)	8 ± 3.4 mm (*n* = 15)	6.1 ± 2.9 mm	9 (43%)	14 (67%)	–	High
Norris et al. [48]	15–61 Y	11 mth	11.9 ± 4.7 mm (*n* = 7)	–	–	5 (63%)	–	Medium
Tos et al. [25]	30 Y (17–45 Y)	51 mth	–	–	8 (100%)	7 (88%)	–	High
Pereira et al. [38]	A:37.3 Y (18–64 Y); B:38.3 Y (13–72 Y)	A: 6–40 mth; B: 8–64 mth	A:5.4 ± 2.6 mm B:11 ± 5.9 mm	–	–	A:12 (100%) B:23 (79%)	0–30 mth	High
Laveaux et al. [49]	47 Y (17–75 Y)	50 mth (11–106 mth)	10.8 ± 2 mm (*n* = 11)	–	6 (50%)	11 (92%)	< 24 h	High
Lee and Shieh [50]	32.3 Y (19–52 Y)	8.58 Y (2.75–12 Y)	4.7 ± 1.2 mm	4 ± 1 mm	–	2 (67%)	17 days–2 years	Medium
Risitano et al. [26]	35 Y (15–70 Y)	> 12 mth	–	–	–	11 (50%)	0–72 hs	High
Tang et al. [51]	23.3 Y (15–34 Y)	2 Y (18–33 mth)	5.1 ± 1.5 mm (*n* = 11)	–	–	11 (61%)	–	Medium
Walton et al. [52]	32 Y (15–55 Y)	13.6 mth (14 mth–2 Y)	4.5 ± 1.5 mm (*n* = 12)	–	9 (50%)	12 (67%)	1 week-9 mth	High
Chiu and Strauch [54]	37 Y (19–61 Y)	27.4 mth (6–72 mth)	A:7.4 ± 1.54 mm; B:11.1 ± 3.4 mm;C:9.0 ± 1 mm	A:5.7 ± 1.7 mm; B:6.5 ± 2.56 mm; C:5.78 ± 2.38 mm	–	A:12 (100%); B:8 (80%); C:4 (100%)	–	Medium
Calcagnotto and Braga Silva [57]	18–45 Y	10.2 ± 1.4 mth	A:8 mm (6–13 mm) B:10 mm (7–15 mm)	A:6 mm (3–12 mm) B:6 mm (4–14 mm)	–	–	A:3 mth (2–7 mth); B:2 mth (1–4 mth)	High
Alligand-perrin et al. [27]	40 Y (8–79 Y)	25.75 mth (16–39 mth)	10.3 mm (3–22 mm)	9 mm (3–22 mm)	41 (77.5%)	47 (89.5%)	14.3 hs (1–90 hs)	High
Laveaux et al. [28]	A:46 Y (27–75 Y) B:33 Y (13–56 Y)	A:62 mth B:202 mth (> 11 mth)	A: 13.7 ± 4.4 mm B: 10.9 ± 5 mm	A: 9.8 ± 4.3 mm B: 8.1 ± 4.3 mm	A:6 (35%); B:11 (73%)	A:11 (65%); B:12 (80%)	A:1813 day (0–13140 day); B:213 day (0–551 day)	High
Rose et al. [68]	29 Y (18–55 Y)	28.3 mth (8–43 mth)	8.3 ± 3.9 mm (3–11 mm)	5.8 ± 2.5 mm (4–15 mm)	14	12	8.4 mth (1–17 mth)	Medium
Chen et al. [29]	33 Y (20–45 Y)	22 mth, (19–22 mth)	A:6.7 ± 1.3 mm B:9.5 ± 1.4 mm	–	15 (94%) (*n* = 16)	16 (100%) (*n* = 16)	3 h-3.5 mth	High
Li et al. [86]	32 Y (16–63 Y)	16 mth (5–32 mth)	6.8 mm (3–9 mm)	–	–	23 (100%)	2.2 h (1.0–3.5 hs)	High
Chen et al. [58]	A:31.9 Y (17–48 Y); B:31 Y (20–47 Y)	A:25 mth (20–26 mth); B:23 mth (19–27 mth)	A:6.4 ± 1.0 mm B: 9.2 ± 1.8 mm	–	A:21 (100%)	A:21 (100%)	–	Medium
Stang et al. [62]	A:43 ± 13 Y; B:40 ± 15 Y	A:15 ± 8 mths; B:16 ± 11 mths	A:9 ± 5 mm; B:10 ± 2 mm	–	–	A:14 (88%); B:9 (75%)	–	High
Chevrollier et al. [67]	39 Y (18–78 Y)	27 mth (6–56 mth)	8.3 ± 5.8 mm (*n* = 12)	–	9 (56%)	9 (56%)	–	High
Kim et al. [69]	33 Y (15–63 Y)	27 mth (24–37 mth)	5.9 ± 0.9 mm	5 ± 0.8 mm	30 (100%)	30 (100%)	–	High
Mcfarlane and Mayer [65]	28 Y (20–59 Y)	7–23 mth	14.9 ± 5.5 mm (*n* = 11)	–	8 (62%)	4 (31%)	1–10 mth	Medium
Nunley et al. [76]	29 Y (16–51 Y)	57 mth (24–89 mth)	8.9 ± 3.6 mm (*n* = 18)		14 (93%) (*n* = 15)		2–11 mth	High
Pilanci et al. [30]	37.5 Y (16–60 Y)	20.7 mth (9.3–41 mth)	7.1 ± 3.3 mm	–	13 (87%)	15 (100%)	50.7 days (9–210 days)	High
Bekir [71]	27 Y (17–38 Y)	35.7 mth	5.9 ± 2.2 mm	–	13 (100%)	13 (100%)	53.3 days	High
Inoue et al. [31]	25.7 Y (18–31 Y)	9.7 mth (6–12 mth)	5.3 ± 1.2 mm	–	–	3 (100%)	–	Medium
Young et al. [55]	27 Y (15–57 Y)	> 6 mth	–	–	–	11 (33%)	< 72 hs	Medium
Meek et al. [32]	31 Y (9–64 Y)	18 mth–10 Y	–	–	–	3 (18%)		High
Acar et al. [75]	33.6 Y (16–60 Y)	14 mth (10–20 mth)	–	–	–	69 (50%)	< 1 day	High
Alghazal et al. [33]	3–70 Y	8–32 mth	–	–	–	80 (91%)	< 1 day	High
Altissimi et al. [34]	35 Y (4—64 Y)	1–7 Y	–	–	–	40 (74%)	< 48 hs	Medium
Efstathopoulos et al. [1]	2–64 Y	–	–	–	–	46 (72%)	< 6 hs	High
Fakin et al. [63]	43 Y (21–77 Y)	42 mth	10.6 ± 4.5 mm	–	–	–	11.1 days	High
Poppen et al. [66]	29 Y (6–67 Y)	10.9 Y (5–13.5 Y)	16.4 ± 11 mm	–	–	47 (96%)	2.8 mth (0–14 mth)	High
Sladana et al. [72]	16–70 Y	30 mth	–	–	–	59 (31%)	< 48 hs	High
Sullivan [35]	20–65 Y	13 mth (6 mth–8 Y)	9.6 ± 4.3 mm (*n* = 33)	–	–	32 (74%)	0–22 mth	High
Bulut et al. [73]	36.4 Y (11–62 Y)	21.4 mth (6–56 mth)	–	–	69 (72%)	87 (91%)	11.7 days (0–150 days)	High
Oruç et al. [59]	A:35.5 Y (15–62 Y) B:41.2 Y (33–53 Y)	> 12 mths A:15.7 mth (12–19 mth)B:17.0 mth (13–19 mth)	A:8.67 ± 1.16 mm; B:9.21 ± 1.25 mm	–	–	A:16 (91.6%) B:9 (85.7%)	A: 1.6 days B: 0 days	High
Young et al. [51]	29 Y (3–67 Y)	10 mth	10 mm	–	–	30 (88%)	< 4 mth	High
Segalman et al. [36]	65 Y (60–72 Y)	> 1 Y	5.5 ± 2.3 mm (*n* = 10)	5 ± 1.6 mm (*n* = 11)	16 (84%)	11 (58%)	–	High
Vahvanen et al. [74]	Average 9.5 Y (young people < 14 Y)	7.5 Y (2–18 y)	–	–	–	18 (100%)	0–14 mth	High
Wang et al. [60]	18–79 Y	> 1 Y	A:6 ± 3.7 mm (*n* = 29); 8 ± 5.7 mm (*n* = 37); B:7 ± 7.4 mm (*n* = 5)	A:3 ± 3.8 mm (*n* = 29); 6 ± 6 mm (*n* = 37); B:4 ± 1.9 mm (*n* = 5)	–	A:64 (84%); B:9 (64%)	–	High
Mennen [84]	38.4 Y (35–42 Y)	> 3 mth	–	–	–	4 (80%)	< 2 Y	High
Voche and Ouattara [37]	30 Y (9–55 Y)	> 9 mth	9.1 ± 1.6 m	7.2 ± 1.9 mm	–	11 (100%)	–	High
Landwehrs and Brüser [70]	52 Y (42–59 Y)	21 mth (11–39 mth)	6 ± 0 mm (*n* = 2)		4 (80%)		–	Medium
Artiaco et al. [64]	45 Y (20–62 Y)	35 mth (8–60 mth)	12.7 ± 3.3 mm	–	7 (100%)	6 (86%)		Medium
Chow and Ng [56]	> 16 Y	3, 6, 12, 18, 24 mth	–	–	–	65 (90%)	–	High

PGA, Polyglycolic acid tubes; M, Man; F, Female; Y, Year; mth, Month; and hs, hours

### Quality assessment and publication bias

All 66 articles were evaluated for the quality assessment using the JBI-MAStARI evaluation tool, and the research evaluation levels were high or medium. The specific evaluation results are shown in Tables 2, 3 and 4. The *P* values derived from Egger’s test indicated their inexistence of the publication bias in most meta-analyses. The results of the Egger test are summarized in Tables 5, 6, 7, 8 and 9.

**Table 3:** Quality appraisal checklist for descriptive/case series (JBI Critical Appraisal Checklist)

Citation	Q1	Q2	Q3	Q4	Q5	Q6	Q7	Q8	Q9	Global quality rating
Kusuhara et al. [8]	N	Y	Y	Y	N/A	Y	Y	Y	Y	High
Mackinnon and Dellon [39]	N	Y	Y	Y	N/A	Y	Y	Y	Y	High
Rinker and Liau [40]	Y	Y	Y	Y	Y	Y	Y	Y	Y	High
Battiston et al. [41]	N	Y	N	Y	Y	Y	Y	Y	Y	High
Neubrech et al. [42]	N	Y	Y	Y	Y	Y	Y	Y	Y	High
Bushnell et al. [16]	N	Y	Y	Y	N/A	Y	Y	Y	Y	High
Lohmeyer et al. [43]	N	Y	Y	Y	N/A	Y	Y	Y	Y	High
Lohmeyer et al. [44]	N	Y	Y	Y	N/A	Y	Y	Y	Y	High
Schmauss et al. [9]	Y	Y	Y	Y	N/A	Y	Y	Y	Y	High
Taras et al. [17]	Y	Y	Y	Y	N/A	Y	Y	Y	Y	High
Arnaout et al. [18]	N	Y	Y	Y	N/A	Y	Y	Y	Y	High
Thomsen et al. [19]	N	Y	Y	Y	N/A	Y	Y	Y	Y	High
Means et al. [20]	Y	Y	Y	Y	Y	Y	Y	Y	Y	High
Rbia et al. [21]	N	Y	Y	Y	Y	Y	Y	Y	Y	High
Guo et al. [22]	N	Y	Y	Y	N/A	Y	Y	Y	Y	High
Ingari [45]	Y	Y	Y	Y	N/A	Y	Y	Y	Y	High
Rinker [46]	Y	Y	Y	Y	N/A	Y	Y	Y	Y	High
Taras [23]	Y	Y	Y	Y	N/A	Y	Y	Y	Y	High
Karabekmez et al. [24]	N	Y	N	Y	N/A	Y	Y	Y	Y	Medium
He [53]	Y	Y	Y	Y	Y	Y	Y	Y	Y	High
Ignazio [47]	N	Y	Y	Y	N/A	Y	Y	Y	Y	High
Norris et al. [48]	N	Y	N	Y	N/A	Y	Y	Y	Y	Medium
Tos et al. [25]	N	Y	Y	Y	N/A	Y	Y	Y	Y	High
Pereira et al. [38]	N	Y	N	Y	Y	Y	Y	Y	Y	High
Laveaux et al. [49]	Y	Y	Y	Y	N/A	Y	Y	Y	Y	High
Lee and Shieh [50]	N	Y	N	Y	N/A	Y	Y	Y	Y	Medium
Risitano et al. [26]	Y	Y	Y	Y	N/A	Y	Y	Y	Y	High
Tang et al. [51]	N	Y	N	Y	N/A	Y	Y	Y	Y	Medium
Walton et al. [52]	N	Y	Y	Y	N/A	Y	Y	Y	Y	High
Chiu and Strauch [54]	N	Y	N	Y	N/A	Y	Y	Y	Y	Medium
Calcagnotto and Braga Silva [57]	Y	Y	Y	Y	Y	Y	Y	Y	Y	High
Alligand-perrin et al. [27]	N	Y	Y	Y	N/A	Y	Y	Y	Y	High
Laveaux et al. [28]	Y	Y	Y	Y	Y	Y	Y	Y	Y	High
Rose et al. [68]	N	Y	N	Y	N/A	Y	U	Y	Y	Medium
Chen et al. [29]	N	Y	Y	Y	Y	Y	Y	Y	Y	High
Li et al. [86]	N	Y	N	Y	Y	Y	Y	Y	Y	High
Chen et al. [58]	N	Y	N	Y	N/A	Y	U	Y	Y	Medium
Stang et al. [62]	Y	Y	Y	Y	Y	Y	Y	Y	Y	High
Chevrollier et al. [67]	N	Y	Y	Y	N/A	Y	Y	Y	Y	High
Kim et al. [69]	N	Y	Y	Y	N/A	Y	Y	Y	Y	High
Mcfarlane and Mayer [65]	N	Y	N	Y	N/A	Y	Y	Y	Y	Medium
Nunley et al. [76]	N	Y	Y	Y	N/A	Y	Y	Y	Y	High
Pilanci et al. [30]	N	Y	Y	Y	N/A	Y	Y	Y	Y	High
Bekir [71]	N	Y	Y	Y	N/A	Y	Y	Y	Y	High
Inoue et al. [31]	N	Y	N	Y	N/A	Y	N/A	Y	Y	Medium
Young et al. [55]	N	Y	N	Y	N/A	Y	Y	Y	Y	Medium
Meek et al. [32]	N	Y	Y	Y	N/A	Y	Y	Y	Y	High
Acar et al. [75]	N	Y	Y	Y	N/A	Y	Y	Y	Y	High
Alghazal [33]	N	Y	Y	Y	N/A	Y	Y	Y	Y	High
Altissimi et al. [34]	N	Y	N	Y	N/A	Y	Y	Y	Y	Medium
Efstathopoulos et al. [1]	N	Y	Y	Y	N/A	Y	Y	Y	Y	High
Fakin et al. [63]	Y	Y	Y	Y	N/A	Y	Y	Y	Y	High
Poppen et al. [66]	N	Y	Y	Y	N/A	Y	Y	Y	Y	High
Sladana [72]	N	Y	Y	Y	N/A	Y	Y	Y	Y	High
Sullivan [35]	N	Y	Y	Y	N/A	Y	Y	Y	Y	High
Bulut et al. [73]	N	Y	Y	Y	N/A	Y	Y	Y	Y	High
Oruç et al. [59]	Y	Y	Y	Y	Y	Y	Y	Y	Y	High
Young et al. [51]	Y	Y	Y	Y	Y	Y	Y	Y	Y	High
Segalman et al. [36]	N	Y	Y	Y	N/A	Y	Y	Y	Y	High
Vahvanen et al. [74]	N	Y	Y	Y	N/A	Y	Y	Y	Y	High
Wang et al. [60]	N	Y	Y	Y	Y	Y	Y	Y	Y	High
Mennen [84]	N	Y	Y	Y	N/A	Y	Y	Y	Y	High
Voche and Ouattara [37]	N	Y	Y	Y	N/A	Y	Y	Y	Y	High
Landwehrs and Brüser [70]	N	Y	N	Y	N/A	Y	Y	Y	Y	Medium
Artiaco et al. [64]	N	Y	N	Y	N/A	Y	Y	Y	Y	Medium
Chow and Ng [56]	N	Y	Y	Y	N/A	Y	Y	Y	Y	High

Y, Yes; N, No; U, Unclear; and N/A, Not applicable

**Table 4:**
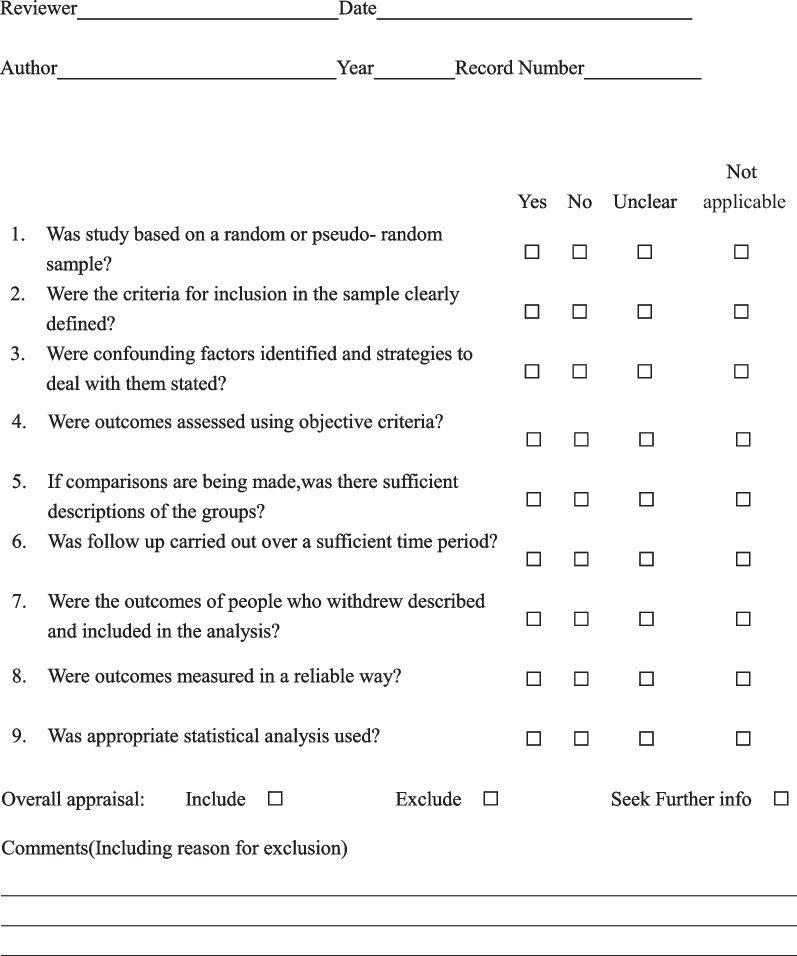
JBI Critical Appraisal Checklist for descriptive/case series

**Table 5:** Summary of static 2-point discrimination results for each repair technique (results of the Egger test, the heterogeneity test, and the meta-analysis)

Repair type	No. of studies	Results of the meta-analyses [S2PD (mm)]	Egger test		Heterogeneity test			
			*t*	*p*	*I*^2^ (%)	*τ*^2^	*p*	Model
Artificial conduit: Polyglycolic acid	4	6.71 (95% CI 4.46; 8.96)	− 0.10381	0.9268	97	4.4801	< 0.01	Random effects model
Artificial conduit: Collagen	8	8.10 (95% CI 6.15; 10.06)	1.6437	0.1513	88	6.2381	< 0.01	Random effects model
Nerve allograft	7	7.88 (95% CI 6.32; 9.43)	1.4158	0.216	96	4.1020	< 0.01	Random effects model
Autograft repair: muscle-in-vein graft	3	8.07 (95% CI 5.02, 11.12)	2.0577	0.288	85	5.9217	< 0.01	Random effects model
Autograft repair: Vein graft	8	8.33 (95% CI 6.13; 10.52)	2.0475	0.08654	96	9.1860	< 0.01	Random effects model
Autologous nerve graft	18	8.46 (95% CI 7.41; 9.50)	1.6997	0.1085	93	4.0666	< 0.01	Random effects model
End-to-end coaptation	11	8.80 (95% CI 7.63; 9.97)	0.10582	0.918	91	3.2487	< 0.01	Random effects model
End-to-side coaptation	4	8.28 (95% CI 6.69; 9.88)	3.9363	0.05889	94	2.2643	< 0.01	Random effects model

**Table 6:** Summary of modified Highet classification good rate for each repair technique (results of the Egger test, the heterogeneity test, and the meta-analysis)

Repair type	No. of studies	Results of the meta-analyses [Highet score]	Egger test		Heterogeneity test			
			*t*	*p*	*I*^2^ (%)	*τ*^2^	*p*	Model
Artificial conduit: Polyglycolic acid	3	0.74 (95% CI 0.53; 0.91)	− 0.2407	0.8496	66	0.0222	0.05	Random effects model
Artificial conduit: Collagen	9	0.83 (95% CI 0.67; 0.95)	0.017577	0.9865	81	0.0537	< 0.01	Random effects model
Nerve allograft	6	0.78 (95% CI 0.66; 0.88)	3.5307	0.02422	68	0.0142	< 0.01	Random effects model
Autograft repair: muscle-in-vein graft	4	0.83 (95% CI 0.58; 0.99)	0.35211	0.7584	66	0.0411	0.03	Random effects model
Autograft repair: Vein graft	8	0.77 (95% CI 0.61; 0.90)	− 0.53158	0.6141	72	0.0345	< 0.01	Random effects model
Autologous nerve graft	14	0.84 (95% CI 0.66; 0.97)	− 0.14966	0.8835	90	0.1186	< 0.01	Random effects model
End-to-end coaptation	18	0.79 (95% CI 0.68, 0.88)	2.8386	0.01186	94	0.0613	< 0.01	Random effects model
End-to-side coaptation	4	0.98 (95% CI 0.85, 1.00)	− 3.8032	0.0627	37	0.0142	0.19	Random effects model

**Table 7:** Summary of moving 2-point discrimination results for each repair technique (results of the Egger test, the heterogeneity test, and the meta-analysis)

Repair type	No. of studies	Results of the meta-analyses [M2PD (mm)]	Egger test		Heterogeneity test			
			*t*	*p*	*I*^2^ (%)	*τ*^2^	*p*	Model
Artificial conduit	5	5.84 (95% CI 4.16, 7.51)	2.8297	0.0662	95	3.0693	< 0.01	Random effects model
nerve allograft	4	5.82 (95% CI 4.51, 7.12)	0.7727	0.5205	88	1.5211	< 0.01	Random effects model
autograft repair (muscle-in-vein graft, vein graft)	7	7.06 (95% CI 5.58, 8.54)	2.4314	0.05928	86	3.3283	< 0.01	Random effects model
autologous nerve graft	6	5.53 (95% CI 4.52, 6.55)	1.1836	0.3021	52	0.7346	0.06	Random effects model
neurorrhaphy	4	4.91 (95% CI 3.72, 6.09)	− 0.28731	0.8009	73	1.0204	0.01	Random effects model

**Table 8:** Summary of Semmes–Weinstein monofilament testing good rate for each repair technique (results of the Egger test, the heterogeneity test, and the meta-analysis)

Repair type	No. of studies	Results of the meta-analyses [SWMF]	Egger test		Heterogeneity test			
			*t*	*p*	*I*^2^ (%)	*τ*^2^	*p*	Model
Artificial conduit	5	0.64 (95% CI 0.28, 0.94)	1.7468	0.179	89	0.1376	< 0.01	Random effects model
Nerve allograft	6	0.86 (95% CI 0.73, 0.96)	− 1.3529	0.2475	68	0.0200	< 0.01	Random effects model
Autograft repair (muscle-in-vein graft, vein graft)	6	0.61 (95% CI 0.40, 0.80)	− 0.45685	0.6715	79	0.0466	< 0.01	Random effects model
Autologous nerve graft	10	0.91 (95% CI 0.80, 0.99)	− 1.7598	0.1165	75	0.0438	< 0.01	Random effects model
Neurorrhaphy	5	0.87 (95% CI 0.73, 0.97)	0.026774	0.9803	77	0.0216	< 0.01	Random effects model

**Table 9:** Summary of all the data in the 4 outcome indicators (Results of the Egger test, the heterogeneity test, and the meta-analysis)

Outcome indicators	No. of studies	Results of the meta-analyses	Egger test		Heterogeneity test			
			*t*	*p*	*I*^2^ (%)	*τ*^2^	*p*	Model
S2PD (mm)	51	8.18 (95% CI 7.66, 8.70)	2.8485	0.005952	94	3.6328	< 0.01	Random effects model
M2PD (mm)	19	5.90 (95% CI 5.34, 6.46)	3.5872	0.001358	89	1.6864	< 0.01	Random effects model
Highet score (%)	61	0.80 (95% CI 0.74, 0.86)	2.6945	0.009205	88	0.0545	< 0.01	Random effects model
SWMF (%)	29	0.81 (95% CI 0.72, 0.88)	− 1.012	0.3196	85	0.0547	< 0.01	Random effects model

### Synthesis of results

All the data extracted from the literature are shown in Table 2. The S2PD, Highet score, M2PD, and SWMF sensory results are summarized in Tables 5, 6, 7 and 8.

**Fig. 2:**
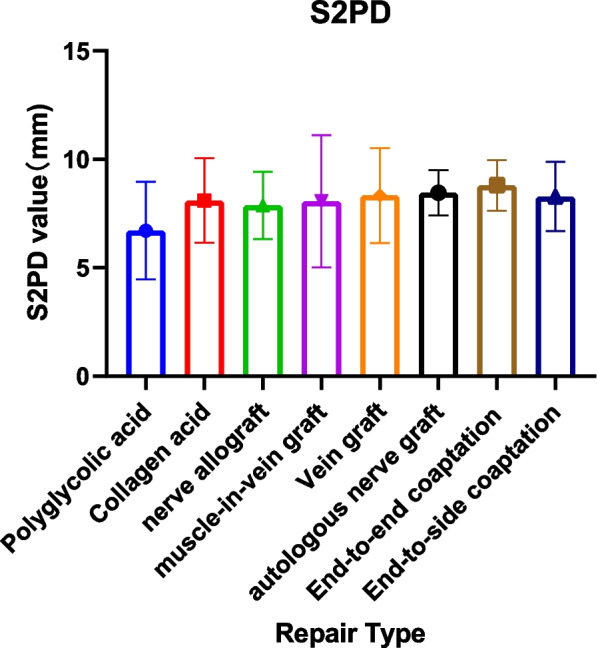
Static 2-point discrimination results for each repair technique

**Fig. 3:**
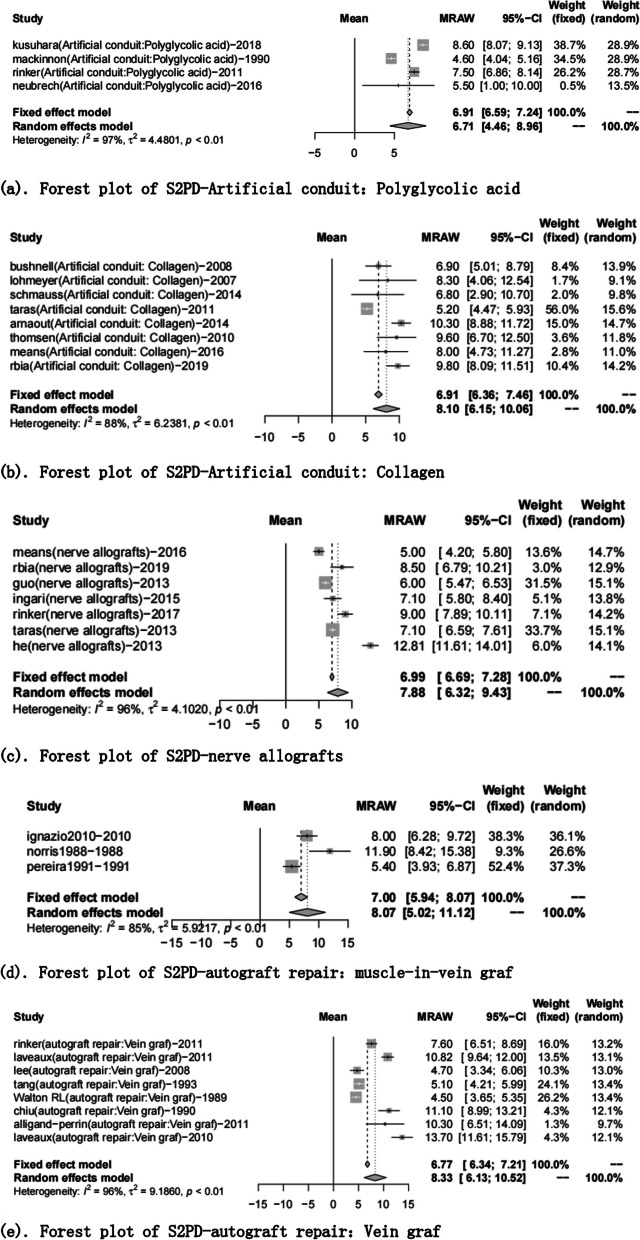

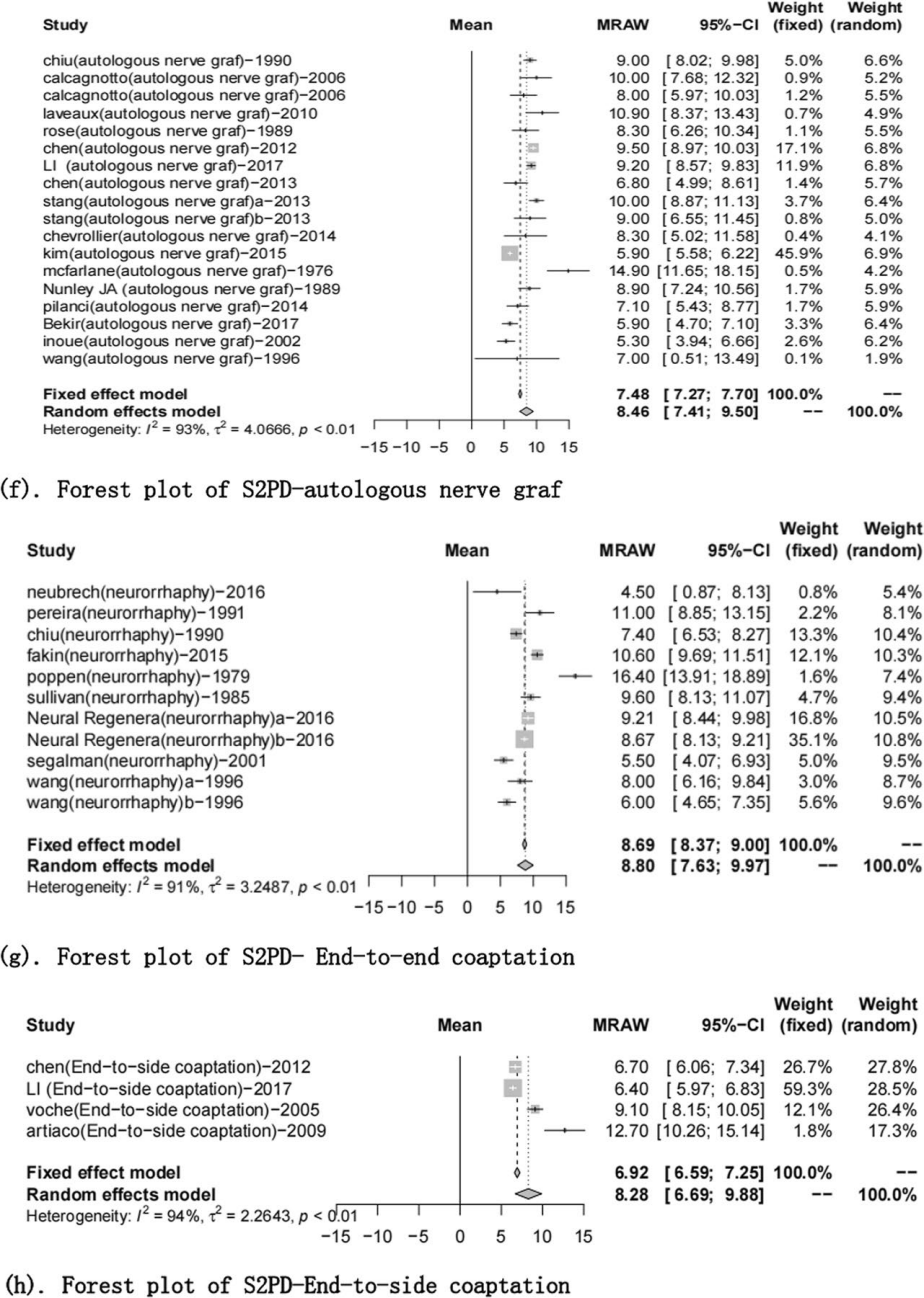
Forest plot of static 2-point discrimination results for each repair technique. **a** Forest plot of S2PD—Artificial conduit: polyglycolic acid; **b** Forest plot of S2PD—Artificial conduit: collagen; **c** Forest plot of S2PD—nerve allografts; **d** Forest plot of S2PD—autograft repair: muscle-in-vein graft; **e** Forest plot of S2PD—autograft repair: vein graft; **f** Forest plot of S2PD—autologous nerve graft; **g** Forest plot of S2PD—end-to-end coaptation; and **h** Forest plot of S2PD—end-to-side coaptation

A total of 51 articles reported the S2PD data [8, 9, 16–24, 27–31, 35–40, 42, 44–71, 76, 86]. After a summary analysis, the polyglycolic acid conduit group was 6.71 mm (95% CI 4.46; 8.96), which was the smallest discrimination distance, the end-to-end coaptation group was 8.80 mm (95% CI 7.63; 9.97), and the postoperative discrimination distance was the largest. The values of the other groups were distributed between them, but they have yet to reach excellent (2–6 mm), just at the good level (7–15 mm) (Table 5, Figs. 2, 3).

**Fig. 4:**
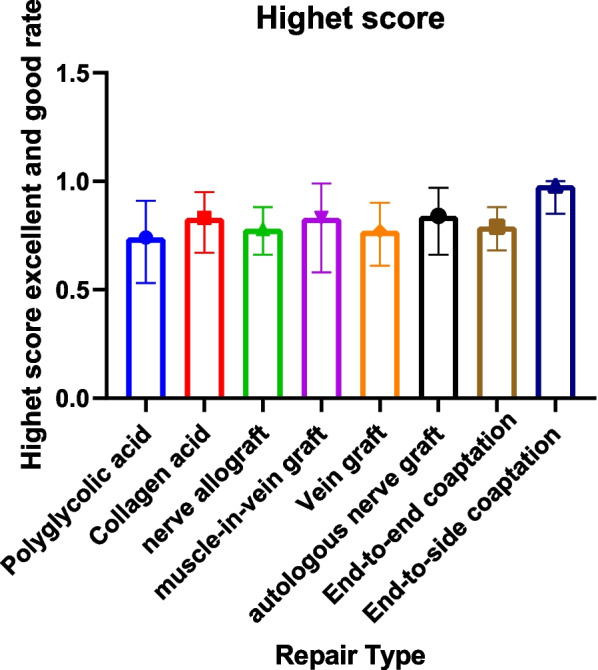
Modified Highet classification good rate for each repair technique

**Fig. 5:**
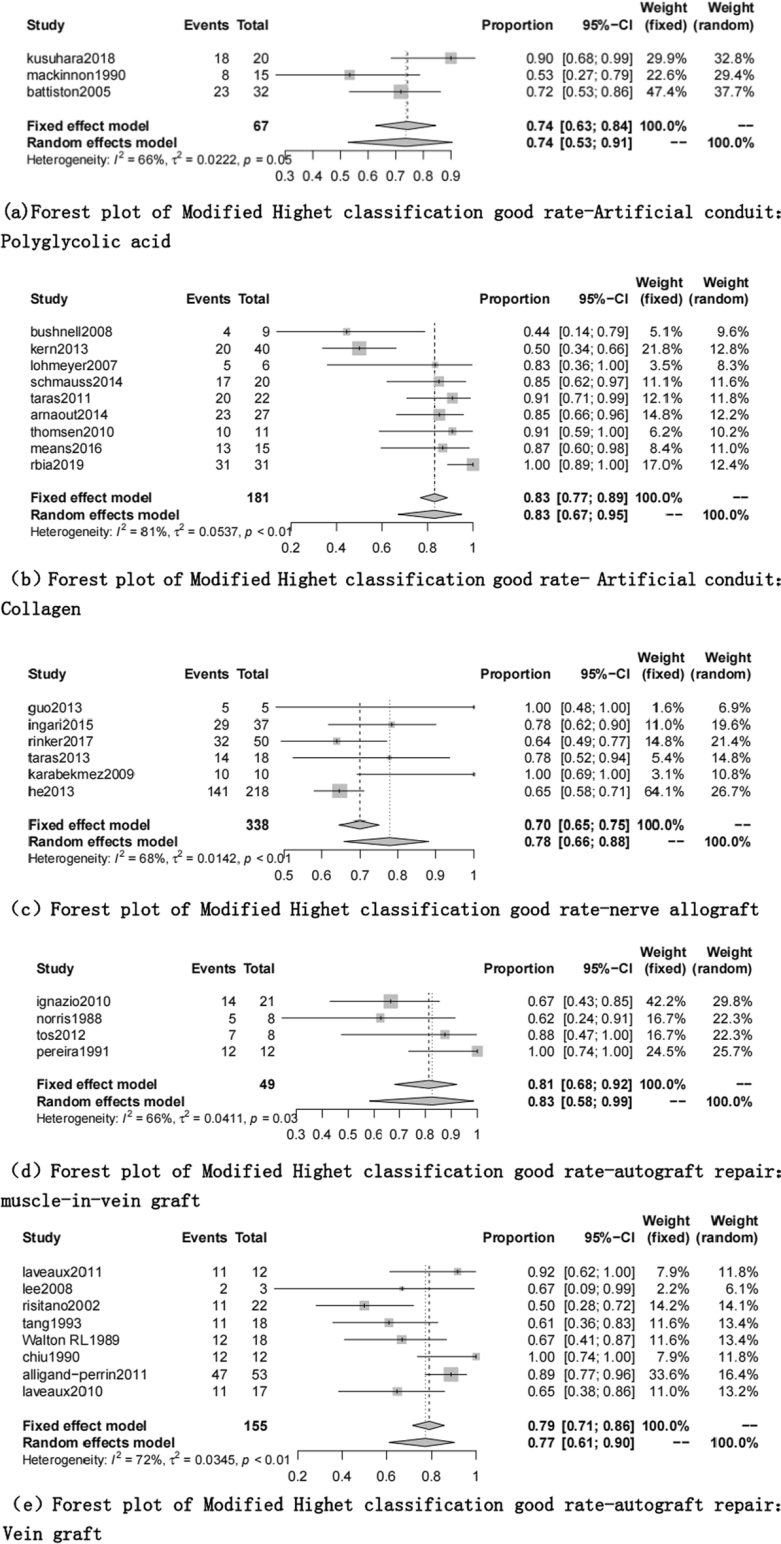

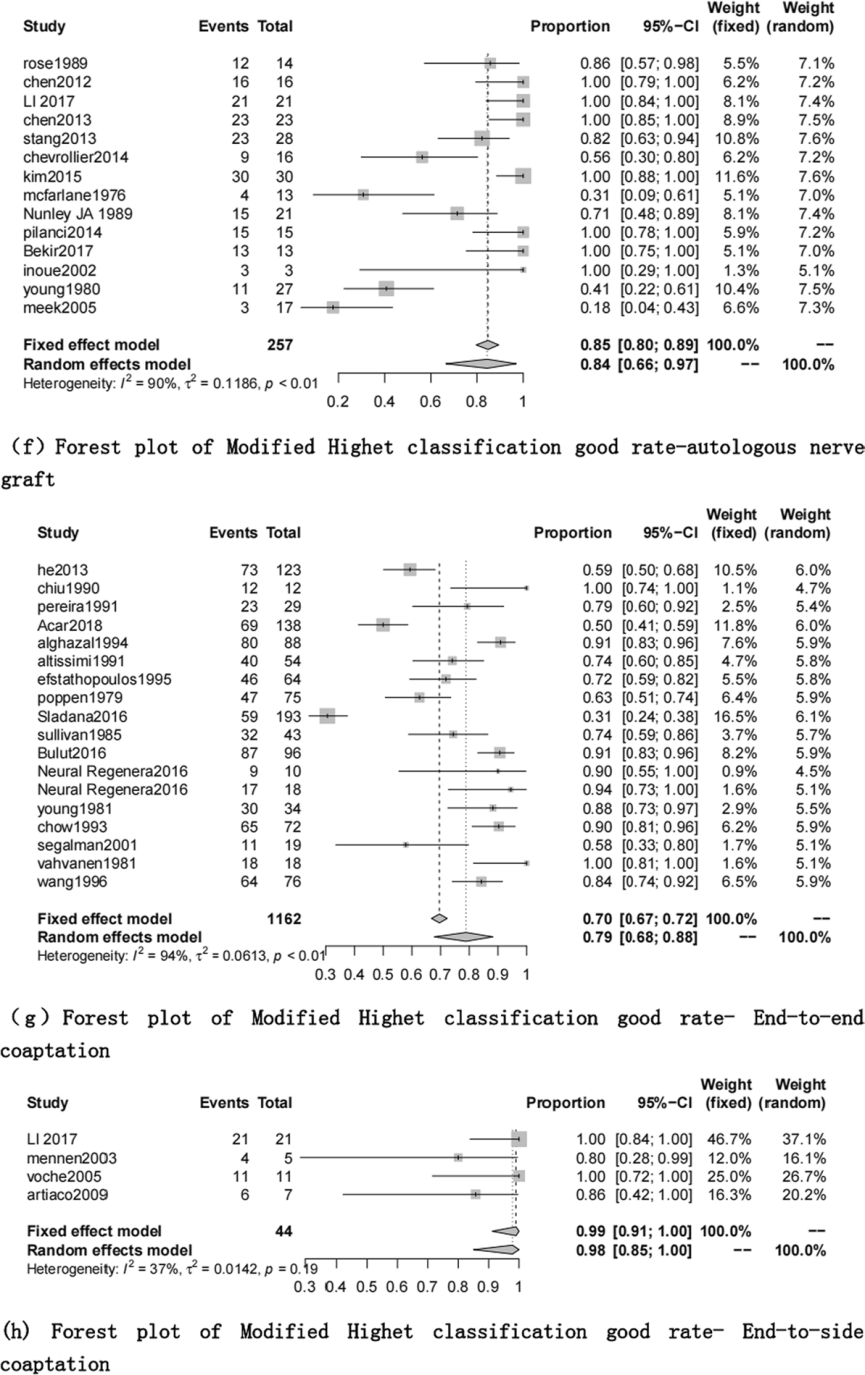
Forest plot of modified Highet classification good rate for each repair technique. **a** Forest plot of modified Highet classification good rate—Artificial conduit: polyglycolic acid; **b** Forest plot of modified Highet classification good rate—Artificial conduit: collagen; **c** Forest plot of modified Highet classification good rate—nerve allograft; **d** Forest plot of modified Highet classification good rate—autograft repair: muscle-in-vein graft; **e** Forest plot of modified Highet classification good rate—autograft repair: vein graft; **f** Forest plot of modified Highet classification good rate—autologous nerve graft; **g** Forest plot of modified Highet classification good rate—end-to-end coaptation; and **h** Forest plot of modified Highet classification good rate—end-to-side coaptation

The excellent rate of modified Highet’s scoring includes 61 articles [1, 7–9, 16–39, 41, 43–56, 58–62, 64–69, 71–76, 86]. The end-to-side coaptation group was 98% (95% CI 0.85, 1.00), and the postoperative felt the excellent rate was the highest. The polyglycolic acid conduit group was 74% (95% CI 0.53; 0.91), and the excellent rate was the lowest (Table 6, Figs. 4, 5).

**Fig. 6:**
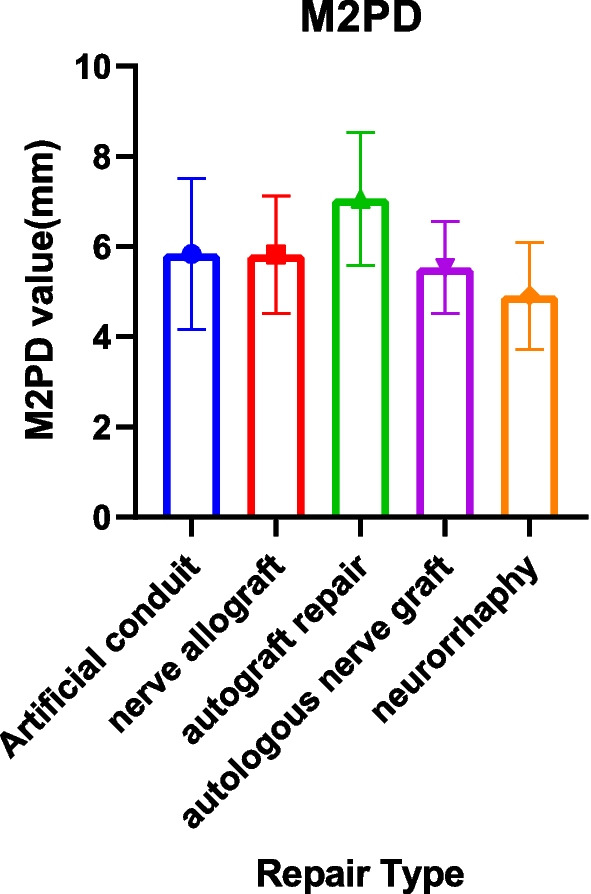
Moving 2-point discrimination results for each repair technique

**Fig. 7:**
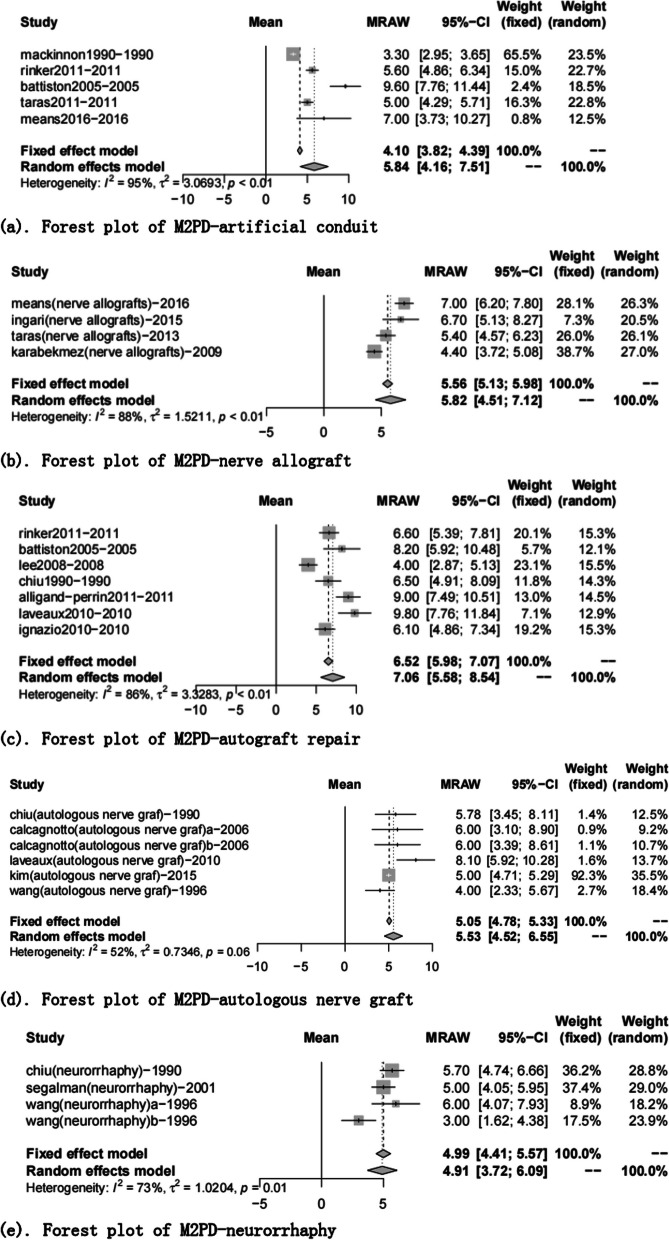
Forest plot of moving 2-point discrimination results for each repair technique. **a** Forest plot of M2PD—artificial conduit; **b** Forest plot of M2PD—nerve allograft; **c** Forest plot of M2PD—autograft repair; **d** Forest plot of M2PD—autologous nerve graft; and **e** Forest plot of M2PD—neurorrhaphy

The M2PD group included 19 articles [17, 20, 23, 24, 27, 28, 36, 37, 39–41, 45, 47, 50, 54, 57, 60, 68, 69]. The neurorrhaphy group was 4.91 mm (95% CI 3.72, 6.09), and the discrimination distance was the smallest; the autograft repair group was 7.06 mm (95% CI 5.58, 8.54), and the postoperative discrimination distance was the largest. The five data sets have yet to reach excellent (2–3 mm) but at a good level (4–7 mm) (Table 7, Figs. 6, 7).

**Fig. 8:**
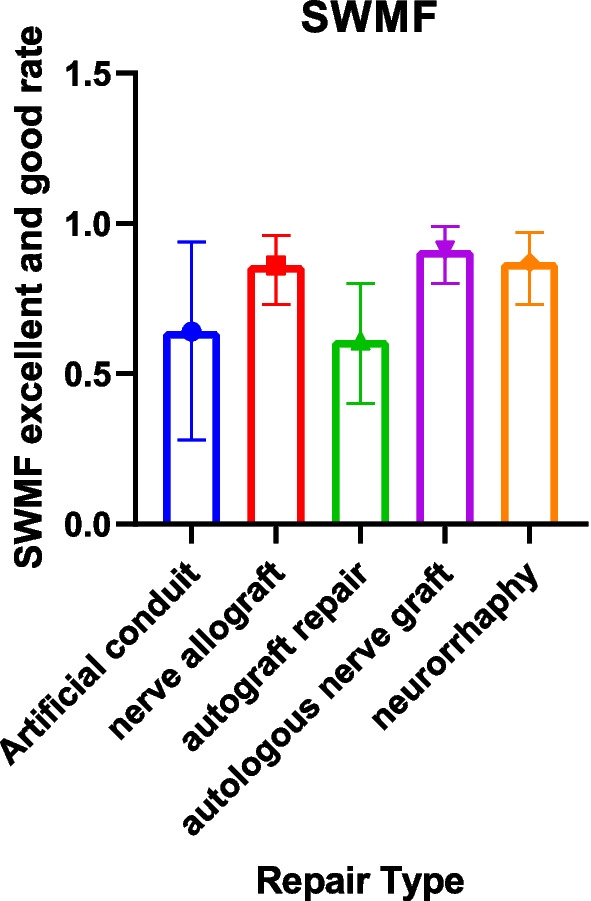
Semmes–Weinstein monofilament testing good rate for each repair technique

**Fig. 9:**
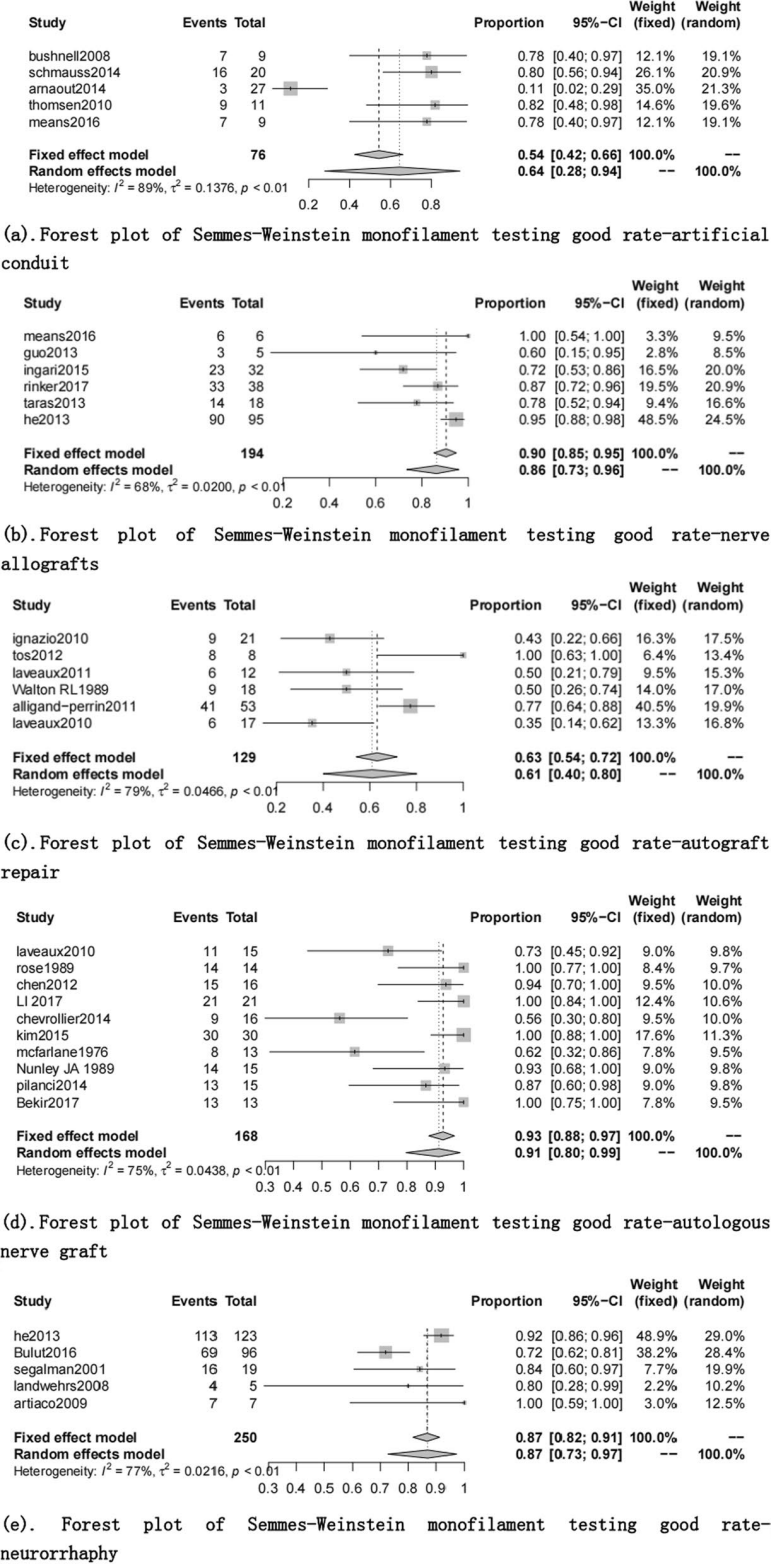
Forest plot of Semmes–Weinstein monofilament testing good rate for each repair technique. **a** Forest plot of Semmes–Weinstein monofilament testing good rate—artificial conduit; **b** Forest plot of Semmes–Weinstein monofilament testing good rate—nerve allografts; **c** Forest plot of Semmes–Weinstein monofilament testing good rate—autograft repair; **d** Forest plot of Semmes–Weinstein monofilament testing good rate—autologous nerve graft; and **e** Forest plot of Semmes–Weinstein monofilament testing good rate—neurorrhaphy

There were 29 documents included in the SWMF data set [9, 16, 18–20, 22, 23, 25, 27–30, 36, 45–47, 49, 52, 53, 64–71, 73, 76, 86]. The highest excellent and good rate was 91% (95% CI 0.80, 0.99) in the autologous nerve graft group. The lowest was 61% (95% CI 0.40, 0.80) in the autograft repair group (Table 8, Figs. 8, 9).

**Fig. 10:**
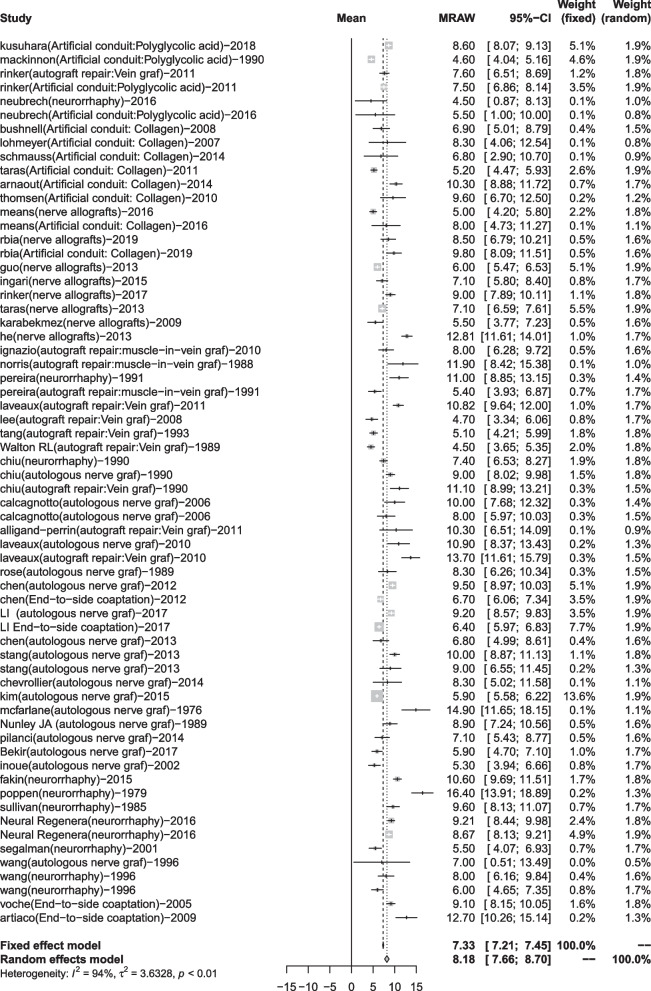
Forest plot of static 2-point discrimination results

**Fig. 11:**
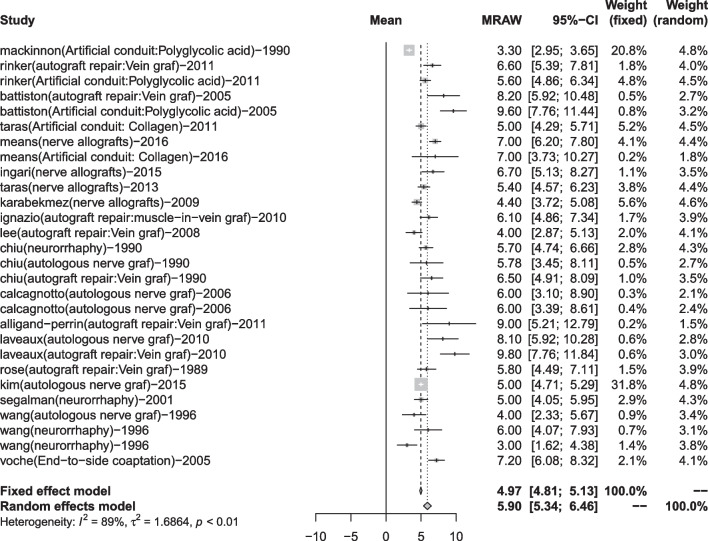
Forest plot of moving 2-point discrimination results

**Fig. 12:**
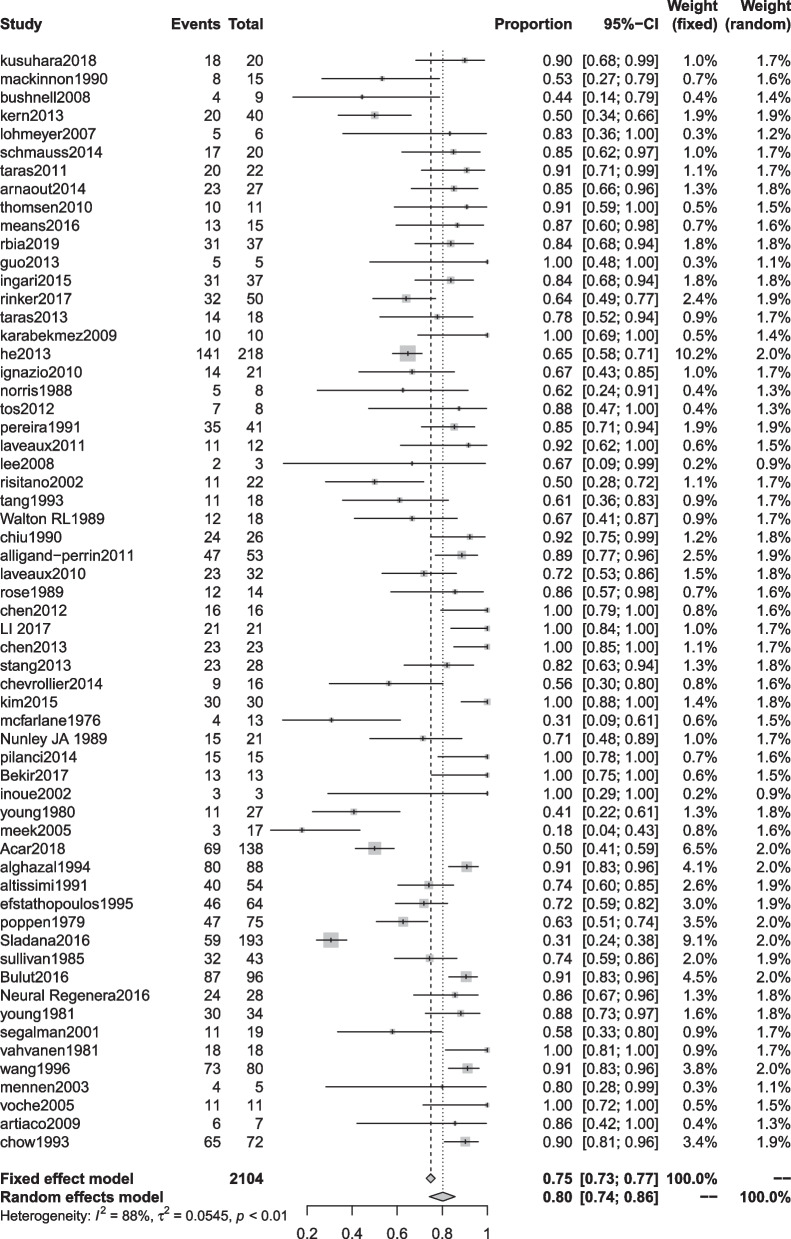
Forest plot of modified Highet classification good rate

**Fig. 13:**
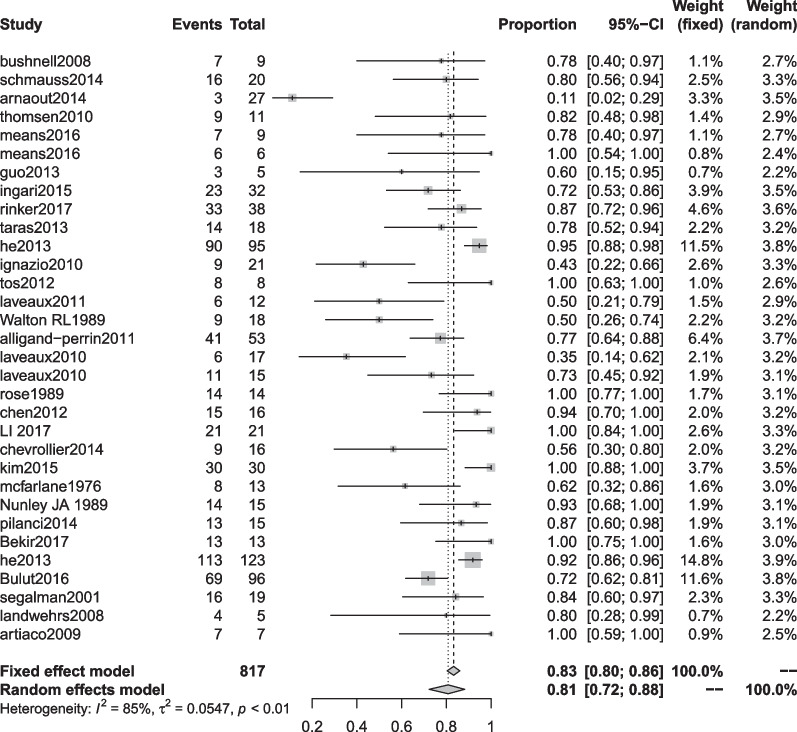
Forest plot of Semmes–Weinstein monofilament testing good rate

Finally, we conducted a summary analysis of all the data in the 4 outcome indicators. S2PD was 8.18 mm (95% CI 7.66, 8.70), M2PD was 5.90 mm (95% CI 5.34, 6.46), Highet score excellent and good rate was 80% (95% CI 0.74, 0.86), and SWMF excellent and good rate was 81% (95% CI 0.72, 0.88) (Table 9, Figs. 10, 11, 12, 13).

We extracted data from 25 articles for statistical analysis in subgroups by injury type. In terms of S2PD values, there was no significant difference in sharp and blunt injuries among the four surgical methods (*P* > 0.05). In terms of the excellent and good rate, the recovery effect of sharp injury was better than that of blunt injury only in the surgical method of neurorrhaphy (*P* = 0.00004472), and there was no statistical difference in the other methods (Tables 12, 13).

We performed statistics on the analysis of other influencing factors in the included literature and completed a summary analysis of complications. In the study of influencing factors, in terms of age factor, 13 articles considered it to have an impact [1, 21, 32–34, 36, 55, 57, 60, 67, 72–74], and nine assumed it to have no effect [9, 20, 43, 45, 63, 65, 66, 71, 75]. In terms of nerve injury interval, 11 papers were deemed to be influential [9, 21, 26, 40, 43, 44, 51, 52, 71, 72, 74], and five pieces that have no influence [20, 32, 60, 65, 67]; four articles were considered to be compelling, [8, 27, 52, 60], and ten articles were considered to be unaffected by the repair time factor [9, 32, 35, 43, 63, 65, 66, 71, 73, 75]; in terms of smoking factors, three papers were supposed to be affected [33, 40, 73], and four pieces were not affected [9, 43, 45, 63] (Table 10).

**Table 10:** Summary of other factors associated with the outcomes and summary of complications

Article	Intervention and control	Number of digital nerve repairs analyzed	Influencing factors					Postoperative complications				
			Age	Never gap	Type of injury	Repair time	Smoking	Neuroma	Cold sensitivity	Paresthesia	Infection	Pain
Kusuhara et al. [8]	C	20	–	–	Y	Y	–	–	–	–	N	–
Mackinnon and Dellon [39]	C	15	–	–	–	–	–	–	–	–	–	Y
Rinker and Liau [40]	C/AU	56	–	Y	–	–	Y	N	–	–	Y (3)	N
Battiston et al. [41]	C/AU	32	–	–	–	–	–	–	–	–	–	–
Neubrech et al. [42]	C/N	38	–	–	–	–	–	N	N	N	N	N
Bushnell et al. [16]	C	9	–	–	–	–	–	N	N	N	N	N
Lohmeyer et al. [43]	C	40	N	Y	Y	N	N	–	–	–	–	–
Lohmeyer et al. [44]	C	6	–	Y	–	–	–	Y (2)	–	Y	–	–
Schmauss et al. [9]	C	20	N	Y	N	N	N	–	–	Y	–	–
Taras et al. [17]	C	22	–	–	–	–	–	N	–	N	N	N
Arnaout et al. [18]	C	27	–	–	Y	–	–	N	N	N	N	N
Thomsen et al. [19]	C	11	–	–	–	–	–	N	N	N	N	N
Means et al. [20]	C/AL	15	N	N	–	–	–	–	–	–	Y (1)	Y (1)
Rbia et al. [21]	C/AL	37	Y	Y	Y	–	–	Y (1)	–	Y (1)	Y (1)	Y (1)
Guo et al. [22]	AL	5	–	–	–	–	–	–	–	–	N	–
Ingari et al. [45]	AL	37	N	–	N	–	N	–	–	–	Y (1)	–
Rinker et al. [46]	AL	50	–	–	–	–	–	N	N	N	N	N
Taras et al. [23]	AL	18	–	–	–	–	–	–	–	–	N	Y (2)
Karabekmez et al. [24]	AL	10	–	–	––	––	–	–	–	–	N	–
He et al. [53]	AL/N	100/123	–	–	–	–	–	–	–	–	Y (3)	Y (6)
Ignazio et al. [47]	AU	21	–	–	–	–	–	Y2	–	–	–	–
Norris et al. [48]	AU	8	–	–	–	–	–	–	–	–	–	–
Tos et al. [25]	AU	8	–	–	–	–	–	–	–	–	–	–
Pereira et al. [38]	AU/N	12/24	–	–	–	–	–	Y	Y	Y	–	Y
Laveaux et al. [49]	AU	12	–	–	–	–	–	N	Y (3)	Y (1)	–	Y
Lee and Shieh [50]	AU	3	–	–	–	–	–	–	–	–	–	Y
Risitano et al. [26]	AU	22	–	Y	–	–	–	N	–	–	–	–
Tang et al. [51]	AU	18	–	Y	–	–	–	–	–	–	–	–
Walton et al. [52]	AU	18	–	Y	Y	Y	–	–	–	–	–	–
Chiu and Strauch [54]	AU/N	26	–	–	–	–	–	–	–	–	–	–
Calcagnotto and Braga Silva [57]	AU	50	Y	–	N	–	–	Y (5)	N	N	N	N
Alligand-perrin et al. [27]	AU	53	–	–	–	Y	–	N	Y (28)	Y (1)	–	N
Laveaux et al. [28]	AU	32	–	–	–	–	–	–	–	–	–	–
Rose et al. [68]	AU	14	–	–	–	–	–	Y	Y	–	–	–
Chen et al. [29]	AU	16/27	–	–	–	–	–	Y	Y (7)	Y	N	Y (5)
Li et al. [86]	AU	23	–	–	–	–	–	–	–	–	–	–
Chen et al. [58]	AU	21/31	–	–	–	–	–	–	Y (5)	–	–	Y (7)
Stang et al. [62]	AU	28	–	–	–	–	–	Y	–	Y	–	–
Chevrollier et al. [67]	AU	16	Y	N	–	–	–	N	Y (2)	Y7	–	Y
Kim et al. [69]	AU	30	–	–	–	–	–	–	Y	–	Y (1)	–
Mcfarlane and Mayer [65]	AU	13	N	N	–	N	–	–	–	Y	–	–
Nunley et al. [76]	AU	21	–	–	–	–	–	N	N	Y	–	–
Pilanci et al. [30]	AU	15	–	–	–	––	–	N	Y (2)	Y	–	–
Bekir et al. [71]	AU	13	N	Y	–	N	–	–	–	Y (5)	–	–
Inoue et al. [31]	AU	3	–	–	–	–	–	–	–	–	–	–
Young et al. [55]	AU	27	–	–	–	–	–	–	–	–	–	–
Meek et al. [32]	AU	17	Y	N	–	N	–	Y	Y	Y	–	–
Acar et al. [75]	N	138	N	–	N	N	–	N	N	N	N	N
Alghazal [33]	N	88	Y	–	Y	–	Y	–	–	Y	–	–
Altissimi et al. [34]	N	54	Y	–	N	–	–	–	–	–	–	–
Efstathopoulos et al. [1]	N	64	Y	–	N	–	–	–	–	Y	–	–
Fakin et al. [63]	N	93	N	–	N	N	N	Y (2%)	Y	Y	–	N
Poppen et al. [66]	N	74	N	–	N	N	–	–	–	–	–	–
Sladana [72]	N	193	Y	Y	Y	–	–	–	–	–	–	–
Sullivan [35]	N	43	–	–	Y	N	–	N	–	N	–	–
Bulut et al. [73]	N	96	Y	–	N	N	Y	–	–	–	–	–
Oruç et al. [59]	N	28	–	–	–	–	–	–	N	N	N	N
Young et al. [51]	N	34	Y	–	–	–	–	–	–	–	–	–
Segalman et al. [36]	N	19	Y	–	Y	–	–	–	–	––	–	–
Vahvanen et al. [74]	N	18	Y	Y	Y	–	–	–	–	–	–	–
Wang et al. [60]	N	90	Y	N	Y	Y	–	–	–	–	–	–
Mennen [84]	N	5	–	–	–	–	–	N	–	N	–	–
Voche and Ouattara [37]	N	10	–	–	–	–	–	N	Y (3)	–	–	Y (1)
Landwehrs and Brüser [70]	N	5	–	–	–	–	–	–	Y	–	–	Y
Artiaco et al. [64]	N	7	–	–	–	–	–	Y	–	–	–	–
Chow and Ng [66]	N	72	–	–	–	–	–	Y (2)	–	–	–	–

N, Neurorrhaphy; AL, Allograft repair; AU, Autograft repair; C, Conduit repair; Y, Yes; N, No; and Numbers indicate the number of columns

The results of the pooled analysis of complications are shown that there were 12 articles of the literature reporting neuroma [21, 29, 32, 38, 44, 47, 56, 57, 62–64, 68], and 14 cases can be counted (artificial conduit: 2 articles, 3 cases; autograft repair: 7 articles, 7 cases; and nerve sutures: 3 articles, 4 cases); 13 publications reporting cold stimulation [27, 29, 30, 32, 37, 38, 49, 58, 63, 67–70], and 50 cases were counted (autograft repair: 10 articles, 47 cases; nerve sutures: 3 articles, 3 cases); 17 papers reporting paresthesia [1, 9, 21, 27, 29, 30, 32, 33, 38, 44, 49, 62, 63, 65, 67, 71, 76], and 15 cases were counted (artificial conduit: 3 articles, 1 case; autograft repair:11 articles,14 cases; and nerve sutures: 3 articles); 6 articles reporting postoperative infections [20, 21, 40, 45, 53, 69], and 10 cases were counted (artificial conduit: 3 articles, 5 cases; nerve allograft: 2 articles, 4 cases; autograft repair: 1 articles, 1 case); 13 articles reported pain [20, 21, 23, 29, 37–39, 49, 50, 53, 58, 67, 70], and 23 cases were counted (artificial conduit: 2 articles, 1 cases; nerve allograft: 3 articles, 9 cases; autograft repair: 6 articles, 12 cases; and nerve sutures: 2 articles, 1 cases) (Table 10).

We analyzed the maximum extent of neurological defects treated by various surgical methods in the literature. The direct suture is the minimum tension-free suture required to repair the defect within 0.5 cm. The largest defect was repaired by autogenous nerve graft, ranging from 0.5 to 9.0 cm. The end-to-side anastomosis technique had no limitation on the length of the defect and was a method of nerve transplantation or bridging (Table 11).

**Table 11:** The maximum extent of nerve defect treated by various surgical methods in the literature

	Artificial conduit (PGA and Collagen)	Autograft repair: (muscle-in-vein graft and vein graft)	Nerve allograft	Autologous nerve graft	End-to-end coaptation	End-to-side coaptation
Never gap (cm)	0.5–3.0 cm	0.5–3.0 cm	0.5–5.0 cm	0.5–9.0 cm	– (< 0.5 cm) Tension-free nerve coaptation possible	– (No nerve length limitation, a method of nerve replantation and bridging)

## Discussion

It has been reported that among all peripheral nerve injuries, the digital nerves were the most common peripheral nerves injured [77]. In the published literature, there were many ways to repair digital nerve injury. However, the clinical practice of digital nerve repair has been lack of consensus. Thus, we analyzed the published literature on finger nerve injury .

**Table 12:** S2PD grouped data comparison on the type of damage

	Conduit repair (C)		Allograft repair (AL)		Autograft Repair (autologous nerve graft/muscle-in-vein graft/vein graft) (AU)		Neurorrhaphy (N)		Total	
	Number of digital nerve repairs	S2PD mean value	Number of digital nerve repairs	S2PD mean value	Number of digital nerve repairs	S2PD mean value	Number of digital nerve repairs	S2PD mean value	Number of digital nerve repairs	S2PD mean value
		(mm)		(mm)		(mm)		(mm)		(mm)
Sharp injury (A)	39	8.13 ± 4.31	29	6.59 ± 1.99	99	9.44 ± 5.05	67	9.34 ± 4.91	234	8.84 ± 4.54
crush injuries (B)	34	8.51 ± 2.48	30	7.73 ± 1.0	45	8.53 ± 3.81	16	10.5 ± 4.27	125	8.59 ± 3.65
*T* value		1.3421		0.0061342		− 0.94997		− 0.09359		0.13197
*P* value		0.1865		0.9951		0.3511		0.9257		0.8951

**Table 13:** Highet score grouped data comparison on the type of damage

	Conduit repair (C)		Allograft repair (AL)		Autograft repair (autologous nerve graft/muscle-in-vein graft/vein graft) (AU)		Neurorrhaphy (N)		Total	
	Number of digital nerve repairs	Highet score (%)	Number of digital nerve repairs	Highet score (%)	Number of digital nerve repairs	Highet score (%)	Number of digital nerve repairs	Highet score (%)	Number of digital nerve repairs	Highet score (%)
Sharp injury (A)	41/55	74.50%	29/29	100%	107/145	73.80%	153/231	66.20%	330/460	71.70%
Crush injuries (B)	31/45	68.90%	29/30	96.70%	53/70	75.70%	32/81	39.50%	145/226	64.20%
Chi-square value	0.16234	–	※	–	0.018429	–	16.66	–	3.7401	–
*P* value	0.687	–	1	–	0.892		0.00004472	–	0.05312	–

※: This is the exact probability using fish

Using the S2PD and modified Highet’s scoring systems, tension-free end-to-end coaptation was the most common method for nerve repair. We found that compared with the other nerve defect repair methods, it seemed that there was no obvious advantage. Autologous nerve transplantation also showed no absolute advantage. As a new material to repair nerve defects, allogeneic nerves have been widely used. Compared with the autologous nerves, it has no obvious advantages. However, it can avoid other postoperative complications caused by nerve extraction and has the same effect as autologous nerve in nerve regeneration. There were some differences between PGA tubes and collagen tubes. In 2003, Laroas et al. published their results on 28 PGA-conduit repairs that with sensory re-education, the success rate could be increased to 100% [78]. In 2007, Waitayawinyu et al. study found better results with collagen conduits than with PGA conduits [79]. Our statistical results showed that there was no significant difference between the two catheters. Vein graft and muscle-in-vein graft as autografts also needed to be obtained from the donor site, but they were not as damaging to the donor site as autologous nerves. The two surgical methods had equivalent results, and there was no absolute advantage when compared with other methods. For large-segment defects or proximal nerve damage, the end-to-side anastomosis technique was an effective method. Its excellent rate was the highest among the 8 methods. Experimental end-to-side nerve suture was first introduced by Kennedy [80], but somehow it was not widely used clinically then. Viterbo et al., the creators of the modern approach of end-to-side neurorrhaphy without harming the donor’s nerve, something that broke paradigm, against all acknowledges, conducted their research by rats, in which they had the peroneal nerve sectioned, the distal ending sutured to the lateral face of the tibial nerve after removing a small epineural window, demonstrating that the anastomosed nerve endings had electrophysiological functions and successfully proving that the end-to-side nerve anastomosis technique was feasible [81–83]. Mennen first reported the use of this technique in humans in 1996 with good results [84]. In the 2003 literature, Mennen reported 56 cases of end-to-side anastomosis, including 5 cases of digital nerve repair, with a good level of neurological functional recovery [7]. Since then, four other scholars have reported related studies, but the number of cases they reported was very small. Recently, new techniques and materials have been used as variants for end-to-side coaptation; however, Geuna S et al. proposed that the bioactive materials as conduits or gene therapy, the role of Schwann cells, and attracting factors derived from the severed trunk should be on the way with further studies [85]. As a new surgical method of nerve repair, there are few studies on the repair of digital nerve. A total of 5 articles [7, 37, 64, 70, 86] and 49 cases were included in our study, and some data could not be extracted. Thus, there may be publication bias.

The data on the excellent rate of SWMF and M2PD of the autograft (muscle-in-vein graft/vein graft) were the worst. These 2 techniques have disadvantages for longer distances such as the collapse of the vein or dispersion of the regenerating axons out of the muscle [47]. We found that none of these methods had significantly different results. Our results were similar as shown in the meta-analysis performed by [11–13].

Through a summary analysis of all the data in the 4 outcome measures, we found that most patients had a good recovery after nerve injury repair. According to the modified Highet classification of nerve recovery, both S2PD and M2PD achieved S3 + or better. The Highet score and SWMF excellent and good rate were all above 80% (Table 1). We found that surgical repair was significantly better than no repair. Our results are consistent with the study performed by Chow et al., which had the same conclusion. [56] In Chow’s literature, 2-year follow-up outcomes were compared between digital nerve repair and no repair. 90% of the 76 patients with nerve repair achieved S3 + or better at 2 years, compared with only 6% of the 36 patients with unrepaired digital nerves. On the other hand, the meta-analysis of Dunlop et al. found that there were little difference between repair and non-repair. The differences in conclusions may be due to different studies included in the analysis [3].

The surgical approach significantly impacts nerve injury and is a critical factor in surgical intervention. The mechanism of injury is another important factor that may affect the degree of damage, the length of nerve defect, the choice of the surgical method, and the outcome of postoperative recovery. Many scholars have researched this factor in the literature included in our study. Kusuhara et al.’s nine studies [8, 18, 21, 33, 43, 52, 60, 72, 74] suggested that the type of injury had an impact on postoperative neurological recovery. Schmauss et al.’s nine studies [1, 9, 34, 45, 57, 63, 66, 73, 75] reported that the type of injury did not affect nerve recovery. We also did a statistical analysis of the data for this factor; through further screening of the included literature, we analyzed 25 kinds of literature with specific injury descriptions. Regarding S2PD value, sharp injury recovered better than blunt injury after four types of surgery, but there was no apparent absolute advantage. In terms of the excellent and reasonable rate, sharp injury has apparent benefits in the recovery of blunt injury after neurorrhaphy, and there is no significant difference between the other three surgical methods. This should be related to the fact that blunt injury can lead to large nerve damage, so only conduit or nerve transplantation can be selected for treatment. After the damaged nerve segment is removed, the nerve stumps become healthy. At this time, there is no significant difference in the effect of the two injury mechanisms on the nerve. However, if the damaged nerve segment is not resected but directly anastomosed, the blunt injury of the nerve is unhealthy and will affect the postoperative recovery. Sharp injury has less damage to the nerve, and the recovery effect after neurorrhaphy is good, while the blunt injury is poor. Therefore, when dealing with blunt nerve injury, the damaged nerve segment should be removed, and the appropriate surgical method should be selected according to the length of the nerve defect.

There are other factors that may affect the postoperative recovery of neuroremediation. In the 5 studies included, it has been shown that age was a factor that affected nerve recovery, especially in children, whose recovery after nerve repair was better than that of adults and the elderly [1, 33, 34, 36, 74]. Repair time, smoking, and follow-up time may have little effect on the recovery after nerve repair. In 2015, a study by Fakin et al. found that the experience of the surgeon was also one of the predicting factors of the outcomes. The repair of the finger artery accompanying the finger nerve had little effect on the postoperative recovery, which was also concluded by Hohendorff et al. [63, 87] In 1985, Sullivan et al. and Murakami et al. found that the number of finger nerve repairs had no difference in the effect of restoration [35, 88]. In a 2016 study done by Bulut et al., it was found that the recovery after finger nerve injury repair was independent of gender and which finger [73]. In 1981, Young et al. compared simple epineurium repair versus perineurium repair, and there was no significant difference in the recovery [55]. In a 2016 study by Sladana et al., it was deemed necessary to use splints after nerve repair [72]. Thomas et al. found that the result of using a microscope was significantly better than using a magnifying glass [89].

Our analysis of the postoperative complications in the included literature found that neuroma, cold stimulation, paresthesia, and pain were the most reported after autograft surgeries. This may be due to the damage to the donor site and poor recovery of the recipient site after transplantation. For complications, the application of allogeneic nerves and nerve conduits was better than autograft.

Our analysis has shown that the length of the nerve defect would affect the postoperative recovery, as well as limit the choice of surgical methods. Of course, we must also consider other factors, such as complications, economic conditions, local hospital technology, repair materials, etc. When there were multiple options to choose from for the optimal repair gap, we had to consider clinical factors associated with recovery when making the decision. There were no significant differences in the outcomes of various surgical methods, and the surgeon should choose a reasonable treatment plan based on the clinical scenario.

There were several limitations of our study. First, the quality of our study is limited by the quality of the included studies, which were mostly case series (level 4 evidence). Second, the strength of our conclusions was limited by the heterogeneous and incomplete outcome data reported across the included studies, and publication bias for the individual studies analyzed. In addition, when analyzing the excellent rate of Highet score, not every study reported outcomes in the same manner. We were forced to use S2PD and M2PD classification systems to group the results into categories that were comparable across sensory outcomes.

## Conclusions

Our study demonstrated that the results of surgical treatment of digital nerve injury are generally satisfactory; however, no nerve repair method has absolute advantages. When choosing a surgical method to repair finger nerve injury, we must comprehensively consider various factors, especially the type of injury, the gap size of the nerve defect, the injury to the patient’s donor site, postoperative complications, the patient’s economic conditions, and the medical level of the local hospital. Whenever tension-free nerve coaptation was possible, end-to-end nerve coaptation was still the method of choice. In the case of nerve defects, the advantages of nerve conduits and allogeneic nerves were relatively high. When the proximal nerve was damaged and could not be connected, the end-to-side anastomosis technique could be selected for bridging to repair. Simultaneously, age, the size of the gap, and the type of injury were also factors that may affect recovery. Certainly, in consideration of the limitations of the study, such as the low qualities, the high heterogeneous, incomplete outcome data reported, and publication bias for the individual studies, conclusions in our study should be interpreted with caution. Therefore, more high-quality randomized controlled studies were definitely needed in order to give a conclusive statement.

## Data Availability

This study included articles which are available via PubMed. All information analyzed in this study was collected in a data set, and this is available from the corresponding author on reasonable request.

## Abbreviations

S2PD: Static 2-point discrimination
M2PD: Moving 2-point discrimination
SWMF: Semmes–Weinstein monofilament testing
PGA: Polyglycolic acid tubes
PRISMA: Preferred Reporting Items for Systematic Reviews and Meta-Analyses
CI: Confidence intervals
JBI-MAStARI: JBI Meta-Analysis of Statistics Assessment and Review Instrument
JBI: Australia’s Joanna Briggs Institute
